# GacA reduces virulence and increases competitiveness in planta in the tumorigenic olive pathogen *Pseudomonas savastanoi* pv. savastanoi

**DOI:** 10.3389/fpls.2024.1347982

**Published:** 2024-02-05

**Authors:** Carla Lavado-Benito, Jesús Murillo, Marta Martínez-Gil, Cayo Ramos, Luis Rodríguez-Moreno

**Affiliations:** ^1^ Área de Genética, Facultad de Ciencias, Universidad de Málaga, Málaga, Spain; ^2^ Instituto de Hortofruticultura Subtropical y Mediterránea “La Mayora”, Consejo Superior de Investigaciones Científicas (IHSM-UMA-CSIC), Málaga, Spain; ^3^ Institute for Multidisciplinary Research in Applied Biology, Universidad Pública de Navarra (UPNA), Edificio de Agrobiotecnología, Mutilva Baja, Spain

**Keywords:** *Pseudomonas savastanoi*, *Pseudomonas syringae*, GacS/GacA two component system, RNA-seq analysis, Gac-Rsm system, woody host

## Abstract

GacS/GacA is a widely distributed two-component system playing an essential role as a key global regulator, although its characterization in phytopathogenic bacteria has been deeply biased, being intensively studied in pathogens of herbaceous plants but barely investigated in pathogens of woody hosts. *P. savastanoi* pv. savastanoi (Psv) is characterized by inducing tumours in the stem and branches of olive trees. In this work, the model strain Psv NCPPB 3335 and a mutant derivative with a complete deletion of gene *gacA* were subjected to RNA-Seq analyses in a minimum medium and a medium mimicking in planta conditions, accompanied by RT-qPCR analyses of selected genes and phenotypic assays. These experiments indicated that GacA participates in the regulation of at least 2152 genes in strain NCPPB 3335, representing 37.9 % of the annotated CDSs. GacA also controls the expression of diverse *rsm* genes, and modulates diverse phenotypes, including motility and resistance to oxidative stresses. As occurs with other *P. syringae* pathovars of herbaceous plants, GacA regulates the expression of the type III secretion system and cognate effectors. In addition, GacA also regulates the expression of WHOP genes, specifically encoded in *P. syringe* strains isolated from woody hosts, and genes for the biosynthesis of phytohormones. A *gacA* mutant of NCPPB 3335 showed increased virulence, producing large immature tumours with high bacterial populations, but showed a significantly reduced competitiveness in planta. Our results further extend the role of the global regulator GacA in the virulence and fitness of a *P. syringae* pathogen of woody hosts.

## Introduction

Two-component regulatory systems (TCSs) play a fundamental role in bacterial detection of extracellular signals and transduction of this information, causing physiological responses that facilitate adaptation to a changing environment (Stock et al., 2000; Gao et al., 2007; Papon and Stock, 2019). TCSs are very common and conserved in bacteria (Wuichet et al., 2010), being the GacS/GacA system one of the most intensively studied. GacS/GacA homologues are widely distributed in Gram-negative bacteria, controlling the expression of numerous genes involved in robust growth as well as a wide range of virulence factors in both animal and plant pathogenic bacteria (Heeb and Haas, 2001; Gooderham and Hancock, 2009; Gauthier et al., 2010; Song et al., 2023). Phytopathogenic bacteria rely on the expression of pathogenicity and virulence factors to cause disease in host plants, and activation of many of them depends on the GacS/GacA pathway (Hrabak and Willis, 1992; Kitten et al., 1998; Chancey et al., 1999; Ferreiro and Gallegos, 2021). However, this system is characterized by displaying a remarkable variability in the pool of regulated genes across different genera, species and pathovars of bacterial plant pathogens.

In *Pseudomonas* species, the GacS/GacA system is initiated by GacS, a histidine kinase located at the plasma membrane that promotes its own phosphorylation after recognition of a yet unknown stimulus. Next, the phosphoryl group is transferred to the cytoplasmic response regulator GacA, which triggers the transcription of a variable number of small non-coding RNAs (sRNAs), among which *rsmX*, *rsmY* and *rsmZ* are the most abundant in *Pseudomonas* and the most intensively investigated (Lapouge et al., 2008; Sobrero and Valverde, 2020). These sRNA molecules modulate the activity of the RsmA family of proteins, which are global posttranscriptional regulators of gene expression in bacteria within the regulatory cascade Gac-Rsm (Sobrero and Valverde, 2020). The RsmA protein has been widely referred to as CsrA, among other names; here, we will adhere to the proposed uniform nomenclature (Sobrero and Valverde, 2020) and use only the Rsm abbreviation for RsmA and homologues. Additionally, in pseudomonads and few other bacteria, the sensor kinases LadS and RetS act as positive or negative regulators, respectively, of the activity of GacS (Sobrero and Valverde, 2020). For the interested reader, several excellent recent reviews include complete descriptions of the Gac-Rsm system (Sobrero and Valverde, 2020; Ferreiro et al., 2021; Song et al., 2023).

The Gac-Rsm system regulates a plethora of processes and phenotypes including the intracellular carbon flux, motility and biofilm formation, pathogenicity and virulence, and biocontrol, among others (Lapouge et al., 2008; Ferreiro and Gallegos, 2021). It has been generally assumed that the regulatory activity of GacA is largely or entirely mediated transcriptionally by direct regulation of the sRNAs and, through the activity of these sRNAs, post-transcriptionally via the Rsm system (Brencic et al., 2009; Zere et al., 2015). However, in *P. aeruginosa* GacA can bind to its own promoter, suggesting autoregulation of its expression, and it can co-regulate the expression of certain genes in collaboration with additional transcription factors, suggesting a complex regulatory network involving transcriptional, posttranscriptional, and translational mechanisms (Huang et al., 2019).

*P. syringae sensu lato* is one of the most important plant-pathogenic bacteria, displaying an outstanding capacity to infect hundreds of species of herbaceous plants, shrubs, and trees (Baltrus et al., 2011; Dillon et al., 2019). In this bacterium, GacS/GacA modulates the expression of many diverse virulence factors and prominently that of the type III secretion system (T3SS), likely the most important pathogenicity determinant of *P. syringae*. The T3SS, encoded by the *hrp*/*hrc* genes, allows bacterial translocation of effector proteins (T3Es) into the host cytoplasm (Alfano and Collmer, 1997; Lindgren, 1997; He, 1998; Hutcheson et al., 2001). However, regulation of the T3SS and virulence by GacS/GacA seems to be strain dependent.

The characterization of the GacS/GacA system in *P. syringae* appears to be deeply biased, being intensively studied in pathogens of herbaceous plants (Hrabak and Willis, 1992; Marutani et al., 2008; Vargas et al., 2013; Ramírez-Zapata et al., 2020) and only reported for a single strain pathogenic to woody hosts, *P. syringae* pv. actinidiae strain A18. A *gacA* mutant of strain A18 showed complete abolition of phaseolotoxin production, and reduction of several virulence-related phenotypes, such as bacterial motility, biofilm formation, production of exopolysaccharide, and HR induction in tobacco plants (Zhang et al., 2018).

*P. savastanoi* pv. savastanoi (Psv) is characterized by inducing hyperplastic growths in the stem and branches of olive trees (Ramos et al., 2012). The pathogenicity of Psv depends on the coordination and expression of several virulence factors (Matas et al., 2012), most notably: production of cytokinins and indole-3-acetic acid (Rodríguez-Moreno et al., 2008; Aragón et al., 2014; Añorga et al., 2020), a Na^+^/Ca^2+^ exchanger (Moretti et al., 2019), quorum sensing (Caballo-Ponce et al., 2018), production of cyclic-di-GMP (Aragón et al., 2015; Martínez-Gil and Ramos, 2018), translocation of type III effector proteins (T3Es) through the T3SS (Pérez-Martínez et al., 2010; Castañeda-Ojeda et al., 2017a; Castañeda-Ojeda et al., 2017b) or the WHOP genomic region (Caballo-Ponce et al., 2017a), related with degradation of phenolic compounds. Psv has become a well-studied model for the mechanisms of pathogenicity in woody hosts and the induction of plant tumours. Here, we aimed to characterize the role of GacA in the pathogenicity/virulence of Psv. For this purpose, we constructed a *gacA* deletion mutant of strain Psv NCPPB 3335 and characterized, by RNA-Seq, the GacA regulon using two different culture media: a standard succinate medium (SSM) and a Hrp inducing medium (HIM), which simulates the apoplastic conditions. Results from this study correlate changes in transcript abundance with diverse virulence-related phenotypes in this bacterial pathogen of woody hosts.

## Experimental procedures

### Bacterial strain, plasmids, and growth conditions

Bacterial strains, plasmids and primers used in this study are summarized in **Supplementary Tables S1**–**S3**, respectively. *P. savastanoi* strains were grown at 28 °C using lysogeny broth (LB) (Lennox, 1955) without glucose and containing 0.5 % NaCl, King’s B (KB) (King et al., 1954), SSM (Meyer and Abdallah, 1978), super optimal broth (SOB) (Hanahan, 1983) or HIM (Huynh et al., 1989) culture media. *Escherichia coli* strains were grown using LB at 37 °C. When required, media were supplemented with appropriate antibiotics at the following final concentrations. For *P. savastanoi*: ampicillin (Ap) (400 μg/mL), gentamicin (Gm) (10 μg/mL), kanamycin (Km) (7 μg/mL), nitrofurantoin (25 μg/mL), and cycloheximide (100 μg/mL). For *E. coli*: Ap (100 μg/mL), Gm (10 μg/mL) and Km (50 μg/mL).

### Construction of *P. savastanoi* mutants and complemented strains

Gene *gacA* was removed from Psv NCPPB 3335 using plasmid p*gacA*-Km, containing the DNA immediately flanking gene *gacA* in both sides (approximately 1.2 kb on each side) separated by an *nptII* (Km)-resistance gene (**Supplementary Tables S2**, **S6**), and following previous procedures (Pérez-Martínez et al., 2007; Matas et al., 2014). Afterwards, the Km gene was removed using the pFLP2 plasmid (**Supplementary Table S2**).

For construction of the complemented strains, the complete coding sequences of *gacA* and *uvrC*, together with their predicted ribosomal binding sites, were amplified from Psv NCPPB 3335 by PCR. After verifying the correct sequence of the resulting amplicons, those fragments were individually cloned under the control of *P_lac_* in pBBR1MCS-5 (Gm^R^) and pBBR1MCS-2 (Km^R^), respectively (**Supplementary Table S2**).

### Genomic organization and gene expression of the *gacA-uvrC* operon in Psv NCPPB 3335

Promoters were predicted with BPROM (Solovyev and Salamov, 2011), a bacterial sigma 70 promoter recognition program (http://www.softberry.com/).

5’ Rapid amplification of complementary DNA (cDNA) ends (RACE) was used to establish the transcription start point and genomic boundary of *uvrC* in Psv NCPPB 3335. Transcripts were obtained from bacterial cultures grown in HIM after 6 hours of induction. 5´ RACE strategy was performed using the 5’/3’ RACE Kit 2^nd^ Generation (Roche Applied Science, Mannheim, Germany). According to the manufacturer’s instructions, three different primers at 357 (SP1), 214 (SP2) and 142 (SP3) base pairs (bp) distance from the ATG of *uvrC* were used for amplification (**Supplementary Table S3**).

### RNA-Seq analysis

To purify RNA, pre-inocula of 20 mL of NCPPB 3335 and Psv-ΔgacA were grown overnight in both KB and SSM media at 28 °C. Bacterial cells from each medium were diluted in fresh KB or SSM to a final optical density at 600 nm (OD_600_) of 0.1, to prepare three biological replicates of 110 mL for each medium and grown at 28 °C with gentle shaking to a final OD_600_ of 0.5 (approximately 5x10^7^ CFUs/mL). Next, bacterial cells from these SSM cultures were collected, each biological replicate resuspended in 12 mL of fresh SSM and divided in twelve 1 mL samples that were frozen in liquid nitrogen and stored at -80 °C. Liquid cultures from each biological replicate grown on KB were pelleted, washed twice with one volume of sterile 10 mM MgCl2, and each resuspended in 110 mL of HIM and incubated at 28 °C for 6 hours. After this, bacterial cells were collected, and each biological replicate resuspended in 12 mL of HIM and divided in twelve 1 mL samples. After a centrifugation step, the pellets were flash-frozen in liquid nitrogen and stored at -80 °C. RNA extractions were performed with the RNAeasy Mini Kit (Qiagen; Hilden, Germany) and following the manufacturer’s instructions. Subsequently, the RNA was cleaned up from traces of genomic DNA using the TURBO DNA-free kit (Invitrogen Corp, CA, USA) following the manufacturer’s instructions. For ribosomal RNA degradation, the Illumina Ribo-Zero Plus rRNA Depletion kit Bacteria (Illumina; CA, USA) was used. The resulting RNA was quantified by spectrophotometry, its integrity assessed by agarose gel electrophoresis, and its quality evaluated on an Agilent Bioanalyzer 2100 using a Pico 6000 RNA bioanalyzer chip (Agilent Technologies, Santa Clara, CA, USA). For genotyping and sequencing, the two biological replicates for each medium with the best quality of purified RNA were used. The TruSeq Stranded mRNA kit (Illumina; CA, USA) was used for genotyping and the Illumina NextSeq550 kit (Illumina; CA, USA) was used for sequencing.

Reads obtained from the RNA-Seq analysis were processed in collaboration with the Ultra sequencing Service of the University of Málaga and the Andalusian Bioinformatics Platform, using a workflow of previously described software packages (Moreno-Pérez et al., 2021). SeqTrimNext (v.2.0.60) was used to clean up and process the sequences, and the FastQC software for quality control; then, sequences were aligned to the reference whole chromosome genome (accession no. CP008742.1) and the three native plasmids of Psv NCPPB 3335 (pPsv48A, FR820585.2; pPsv48B, FR820586.1; pPsv48C, FR820587.2) with the Bowtie2 tool (Langmead and Salzberg, 2012). Transcript abundance, measured in fragments per kilobase per million mapped reads (FPKM), and differential gene expression were calculated using Tuxedo together with the Cufflinks suit of tools (Trapnell et al., 2010; Ghosh and Chan, 2016). CuffDiff was used to identify differentially expressed genes (DEGs). Only those genes with an adjusted P-value (q-value) < 0.05 and a log_2_ fold change ≤ -0.5 or ≥ 0.5 were considered significant. Graphical representation of the differential expression results was carried out using the CummeRbund package in R (Goff et al., 2012).

### RT-qPCR assays

For real-time quantitative PCR (RT-qPCR), DNA-free total RNA samples described in the previous section were used. cDNA synthesis was carried out using random hexamers included in the iScriptTM cDNA synthesis kit (BioRAD, CA, USA) and 1 μg of DNA-free total RNA as template. RT-qPCR primers were designed with Primer3Plus (Untergasser et al., 2007) and Bacon Designer Free (Thornton and Basu, 2015) (**Supplementary Table S3**). Efficiency curve analysis to confirm the amplification specificity of primer pairs was carried out as described (Vargas et al., 2011). The relative transcript abundance was calculated using the *ΔΔ* cycle-threshold (Ct) method (Livak and Schmittgen, 2001). Transcriptional data was then normalized according to the expression of the housekeeping gene *gyrA* by calculating the difference between the relative expression of the analysed gene and that of the *gyrA* gene (ΔΔCt = Ct studied gene – Ct *gyrA*). Fold change values were calculated as 2^-ΔΔCt^ (Pfaffl, 2004; Rotenberg et al., 2006). RT-qPCR analyses were carried out in triplicate with three biological replicates. Change rate is represented by the Psv-ΔgacA expression divided by the wild-type strain expression.

### Bioinformatics

Identification and comparison of homologous *rsm* genes in Psv NCPPB 3335 was carried out by blastp and blastn, using the Geneious 8.1.9 program (Kearse et al., 2012), and the previously described *rsm* sequences in 1448A (Ramírez-Zapata et al., 2020), and Pto DC3000 (Chatterjee et al., 2003; Ferreiro et al., 2018). Sequence alignments were performed using Multalin (http://multalin.toulouse.inra.fr/multalin/) (Corpet, 1988) and Needle servers (https://www.ebi.ac.uk/Tools/psa/emboss_needle/) (Madeira et al., 2022). Additionally, the different *rsm* transcripts were identified using the IGV program by aligning the original.fna sequence with the.gtf and bowtie.bam files extracted from the RNA-Seq analysis.

### Transmission electron microscopy (TEM)

For transmission electron microscopy (TEM) analysis, Psv strains were grown at 28 °C for 48 h on LB. Then, bacteria were collected, washed three times with 10 mM MgCl_2_, and resuspended in SSM medium. After incubation at 28 °C overnight, bacterial cultures were resuspended in SSM to an OD_600_ of 0.1 and incubated with shaking at 28 °C until they reached an OD_600_ of 0.6. Grids were deposited over 30 μL of bacterial suspensions and incubated for 1 h at room temperature. Subsequently, grids were treated with 4 % paraformaldehyde for 10 minutes, washed with water for 5 minutes, negatively stained with 1 % uranyl acetate for 30 seconds and washed once with water for 30 seconds. The grids were allowed to dry for 24 h and imaged on a FEI Tecnai G2 20 TWIN TEM at an accelerating voltage of 80 kV. Images were acquired using TIA FEI Imaging Software v.4.14 (Berlanga-Clavero et al., 2022).

### Swimming motility assay

Psv strains were grown at 28 °C for 48 h on KB in the dark. Cells were resuspended in KB to an OD_600_ of 1 (approximately 1x10^9^ CFUs/mL). Two microliters of the bacterial suspension were spotted onto soft KB agar (KB with 0.3 % agar) and plates were incubated for 72 h at 25 °C and 60 % relative humidity in the dark (Antúnez-Lamas et al., 2009). The surface area of swimming colonies was photographed and quantified using the area selection tool of the image software FiJi (Schindelin et al., 2012).

### Hydrogen peroxide tolerance assay

Bacterial suspensions were prepared to an OD_600_ of 1 (approximately 1x10^9^ CFUs/mL) and 100 μL plated on SSM using a Drigalski plating loop. Thirteen-millimetre diameter Whatman paper discs were wetted with 100 μL of 1.5 % H_2_O_2_ and allowed to dry for 15 minutes. The discs were placed in the centre of each plate and incubated at 28 °C for 48 h before measuring the diameter of the growth inhibition haloes.

### Detection of reactive oxygen species inside live cells

To determine the intrinsic oxidative stress of the cells, the Psv strains were grown overnight in 20 mL of LB at 28 °C. Bacterial cells were diluted 1:10 and disaggregated using a 23-gauge needle. Samples of 100 μL each were incubated with 0.2 μL of 2.5 μM CellROX™ Green Reagent (Invitrogen, Corp, CA, USA) for 30 minutes at 37 °C with shaking. Cells were washed three times with Phosphate Buffered Saline (PBS) and then thoroughly resuspended by passing through a needle (Cámara-Almirón et al., 2020). Five microliters of each sample were dropped on a slide and observed using a Leica SP5 confocal microscope equipped with a HCX PL APO lambda blue 63.0x1.40 OIL UV objective. Images were processed using the Leica Application Suite Advance Fluorescence v.2.7.3.9723 (LCS Lite, Leica Microsystems) and the FIJI/ImageJ software. The laser settings, scanning speed, photomultiplier detector gain, and pinhole aperture were constant for the five images per strain acquired for each of the three experiments performed, and were as described (Berlanga-Clavero et al., 2022).

### Hypersensitivity reaction assay

Two-month-old *Nicotiana tabacum* var. Newdel plants were used for the HR assays. Plants were grown with a photoperiod of 16 h of light, 8 h of darkness at 26 °C. Bacterial suspensions were prepared in 10 mM MgCl_2_ with low population density, 1x10^6^ CFUs/mL. Then, a small wound was made on the abaxial side of the leaf with a hypodermic needle and the bacterial suspension was introduced into the periplasmic space using a 1 mL blunt syringe. The development of symptoms was observed 2 days and 3 days post inoculation (dpi). Images were taken with a high-resolution digital camera (Nikon DXM 1200, Nikon Corp).

### Plant bioassays

Pathogenicity was tested on *Olea europaea* plants, derived from seeds of cv. Arbequina germinated *in vitro*, grown at 60 % humidity and 26 °C. For inoculation, bacterial suspensions were prepared in 10 mM MgCl_2_ to a final OD_600_ of 0.1 (approximately 1x10^7^ CFUs/mL) and two wounds made in the stem of the plant with a scalpel were each inoculated with 5 μL. Four plants were used per strain in each of the three replicate assays. During the 54-day trial, the volume of tumours in each of the inoculated wounds was measured every week. At the end of the trial, symptoms were photographed with a high-resolution digital camera (Nikon DXM 1200). Bacteria were recovered from tumours by grinding them using a mortar and pestle containing 1 mL of sterile 10 mM MgCl_2_. Serial dilutions were plated onto LB supplemented with nitrofurantoin, cycloheximide and the corresponding antibiotic, as described (Penyalver et al., 2006; Pérez-Martínez et al., 2007; Matas et al., 2012).

Competitive index (CI) assays were performed on *in vitro* olive plants obtained from a germinated seed of *O. europaea* cv. Arbequina and grown on DKW medium (Driver and Kuniyuki, 1984) supplemented differently in each of the three culture periods (proliferation, channelling, and maintenance). Plants were inoculated with bacterial suspensions in 10 mM MgCl_2_ in a 1:1 ratio (wild-type strain: mutant strain). A single wound per plant was inoculated with approximately 5x10^3^ CFUs of each strain, using six plants per experiment and repeating the experiment three times (a total of 18 inoculated wounds). After 30 dpi under 25 °C, 50-60 % humidity and 16 h light photoperiod, symptoms were visualized using a stereo microscope (Leica MZ FLIII; Leica Microsystems, Wetzlar, Germany). Bacteria were recovered from tumours as above, and serial dilutions were seeded on LB and LB+Km for colony counting after 2 days at 28 °C. The number of CFUs of the mutant strain was obtained from the colonies that grew on the Km plates, while the number of wild-type bacteria was obtained by subtracting the number of mutant bacteria from the total number of bacteria grown on LB plates (Macho et al., 2007; Macho et al., 2016). The CI is defined as the mutant to wild-type ratio in the output sample divided by the mutant to wild-type ratio in the input (inoculum) sample (Freter et al., 1981; Taylor et al., 1987), the input ratio being close to 1.

### Statistics and reproducibility

Statistical analyses were performed using GraphPad Prism version 9. p-values < 0.05 were considered significantly different. Asterisks indicate the level of statistical significance: *p < 0.05, **p < 0.01, ***p < 0.001 and ****p < 0.0001. When needed, data were normalized to the corresponding internal standard of the samples. All experiments were repeated at least three independent times, with similar results.

## Results

### Genomic conservation of GacS/GacA downstream regulatory elements in Psv NCPPB 3335

*Pseudomonas* species contain two to seven Rsm gene homologues in their genome, with different levels of sequence identity (Sobrero and Valverde, 2020). Genomic analysis of Psv NCPPB 3335 revealed four Rsm homologues (RsmA, RsmC, RsmE and RsmH1) and seven different sRNA genes (*rsmX1*, *rsmX2*, *rsmX3*, *rsmX4*, *rsmX5*, *rsmY* and *rsmZ*) (**Table 1**). The Rsm deduced products and the sRNA genes showed a high sequence identity with their respective homologues in Pto DC3000 (Ferreiro et al., 2018) and Pph 1448A (Ramírez-Zapata et al., 2020) (**Table 1**). Therefore, Psv NCPPB 3335 contains a similar, but distinct complement of Rsm homologues and sRNA genes to other *P. syringae* strains, which should allow for the full functionality of the GacS/GacA regulatory system.

**Table 1:** Characterization of GacS/GacA transcriptional regulation systems in *Pseudomonas savastanoi* pv. savastanoi NCPPB 3335.

	Sequence identity^a^		FPKM^b^				Fold change (log_2_)^c^	
			NCPPB 3335		Psv-△gacA			
	*Ps* pv. tomato	*Ps* pv. phaseolicola	SSM	HIM	SSM	HIM	SSM	HIM
Rsm proteins								
RsmA	100	100	492.65	434.22	679.49	463.82	0.46	0.09
RsmC	94.9	54.3^d^	14.31	22.97	14.11	19.22	- 0.02	- 0.26
RsmE	100	100	533.67	230.10	288.33	353.97	**- 0.89**	**0.62**
RsmH1	94.6	91.9	173.41	1145.59	1266.69	983.77	**2.87**	- 0.22
sRNAs								
*rsmX1*	91.8	100	0	8.85	7.63	15.71	*	0.83
*rsmX2*	96.6	100	36.88	71.32	27.79	37.53	- 0.41	- 0.93
*rsmX3*	98.3	100	194.87	354.95	97.93	288.01	- 0.99	- 0.30
*rsmX4*	96.5	98.2	3.54	55.45	23.54	39.29	2.73	- 0.50
*rsmX5*	96.6	100	92.34	83.99	81.56	142.42	- 0.18	0.76
*rsmY*	91.6	90.8	480.64	1507.22	523.54	373.44	0.12	**- 2.01**
*rsmZ*	94	100	14.38	19.17	17.16	15.39	0.26	- 0.28

a Sequence identity: percent amino acid (Rsm proteins) or nucleotide (small RNAs) identity compared to *Pseudomonas savastanoi* pv. savastanoi NCPPB 3335 performed by global sequence alignment.

b FPKM indicates fragments per kilobase of gene fragments per million reads, in an RNA-Seq analysis.

c Fold change indicates average differential gene expression (log_2_ normalized) between the wild-type strain, NCPPB 3335, and the Psv-△gacA strain in SSM and HIM media. Positive and negative fold change reflect an increased or a decreased level of gene expression in strain Psv-△gacA. Cells with grey shading and bold fold change values indicate significant deregulation (*q* < 0.05). *The number of readings in one of the conditions is 0 so that the fold change cannot be calculated.

d Gene *rsmC* of strain 1448A is truncated due to an insertion of IS*Psy17*, resulting in a shorter chimeric product, hence the lower percentage of identity.

### RNA-Seq analysis

Total RNA was purified from cultures of Psv NCPPB 3335 and its Δ*gacA* mutant grown in SSM, a minimal medium, and HIM medium, which simulates conditions within the plant apoplast and causes the induction of the *hrp* genes (Huynh et al., 1989). Following previous analyses of the role of GacA in *P. syringae* (Chatterjee et al., 2003; Marutani et al., 2008; Ortiz-Martín et al., 2010; Ferreiro et al., 2018), cells were collected during the log phase (OD_600_ of 0.5) in order to better capture the early induction of virulence genes. To compare transcripts abundance, two biological replicates per strain and media were subjected to RNA Illumina sequencing.

In total, 143.2 and 131.0 million high-quality reads (clean reads) were obtained for the SSM and HIM samples, respectively (**Supplementary Tables S4**, **S5**). By iterative sequence alignment, an average of 99.40 % (SSM) and 99.44 % (HIM) of clean reads were mapped to the Psv NCPPB 3335 genome. Out of the 5508 genes annotated in the GenBank record of the Psv NCPPB 3335 chromosome, a total of 5472 genes (99.35 % of the total) were covered by Illumina sequencing in SSM for strain Psv-ΔgacA (**Supplementary Table S6**). In HIM medium, Illumina sequencing covered 99.44 % (5477 genes) and 99.44 % (5476 genes) of the genes in strains NCPPB 3335 and Psv-ΔgacA, respectively (**Supplementary Table S6**). Furthermore, in both media and strains, the Illumina RNA sequencing also covered the 68, 53 and 51 genes annotated in the GenBank records of plasmids pPsv48A, pPsv48B and pPsv48C, respectively (**Supplementary Table S6**).

### Genomic context of gene *gacA* in *P. savastanoi* pv. savastanoi NCPPB 3335

O’Malley and Anderson (2021) showed that deletion of the *gacA* gene in Pto DC3000 did not affect the expression of the downstream genes *uvrC* and *psgA*, which is relevant because of the regulatory activity associated to gene *uvrC* (Humann et al., 2009; O’Malley et al., 2019). This is in contrast with the significant downregulation of *uvrC* observed in our RNA-Seq analysis of strain Psv-ΔgacA (**Table 2**). We therefore sought to analyse the transcriptional organization of these genes in strain NCPPB 3335.

**Table 2:** RNA-Seq analysis expression values of genes *gacS* and *gacA*, and genes downstream of *gacA*, from *Pseudomonas savastanoi* pv. savastanoi NCPPB 3335 in SSM and HIM media.

Gene	FPKM^a^				Fold change^b^ (log_2_)	
	NCPPB 3335		Psv-△gacA			
	SSM	HIM	SSM	HIM	SSM	HIM
*gacS*	53.73	66.45	46.90	48.46	-0.20	-0.46
*gacA*	424.70	444.74	0	0	*	*
*uvrC*	146.91	123.83	4.35	4.42	**-5.08**	**-4.81**
*psgA*	126.52	287.92	75.90	241.85	**-**0.74	-0.25

a FPKM indicates fragments per kilobase of gene fragments per million of readings, in an RNA-Seq analysis.

b Fold change indicates average differential gene expression (log2 normalized) between the wild-type strain and the Psv-△gacA strain in SSM and HIM media. Negative fold change reflect a decreased level of gene expression in strain Psv-△gacA. Cells with grey shading and values in bold indicate genes with a signiﬁcant diﬀerential expression (*q* < 0.05). *The number of readings was 0, because strain Psv-△gacA contains a deletion of the complete *gacA* coding sequence, so that the fold change cannot be calculated.

**Figure 1:**
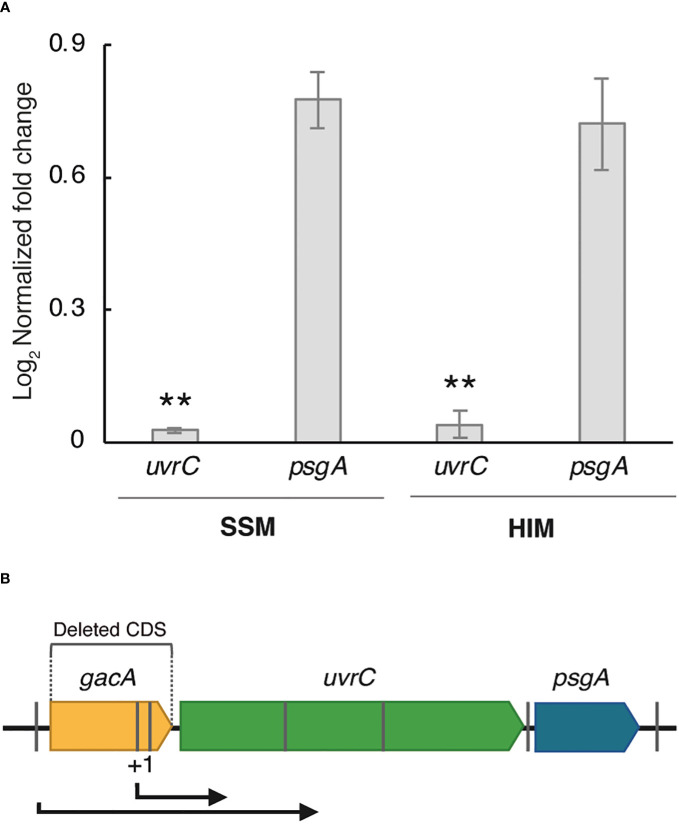
Characterization of the genomic context of gene *gacA*. **(A)** Relative expression of genes *uvrC* and *psgA* in SSM and HIM media by RT-qPCR. Bars represent the average expression values of strain Psv-ΔgacA relative to those of NCPPB 3335, both of which were previously normalized to the constitutive expression of gene *gyrA*. Error bars represent the standard deviation. Asterisks (**) indicate significant differences (Student’s t-test, *p* < 0.01). **(B)** Relative position of genes *gacA*, *uvrC* and *psgA* in the genome of strain NCPPB 3335. Grey bars indicate the approximate location of relevant -35/-10 promoters predicted by BPROM. The short and long bent arrows indicate transcription from, respectively, the transcription start site identified by 5’ RACE (+1) and a predicted promoter, as supported by RNA-Seq.

An RT-qPCR analysis of cells grown in SSM and HIM media showed a significantly reduced expression of *uvrC* in strain Psv-ΔgacA, in agreement with the RNA-Seq data, whereas the expression of gene *psgA* was comparable to that of the wild-type strain (**Figure 1A**). Using BPROM, we identified several -35 and -10 regulatory elements typical of Pribnow-type promoters in the *gacA* DNA region (**Figure 1B**). A 5´ RACE assay with strain NCPPB 3335 showed that the *uvrC* transcription start site (+1) was located 193 bp upstream from its predicted start codon (**Supplementary Figure S1A**). This result confirms that the coding sequence of *gacA* includes a functional promoter for *uvrC*, but upstream of the -35 and -10 regions predicted bioinformatically. However, in strain Psv-ΔgacA we did not observe a complete absence of transcripts covering gene *uvrC* (**Table 2**, **Supplementary Figure S1B**), likely suggesting a low level of *uvrC* transcription from the promoter upstream of gene *gacA* (**Figure 1B**).

Nevertheless, the altered transcription of gene *uvrC* that is associated to certain mutations in *gacA* was shown to confound the assessment of the actual regulatory role of GacA (O’Malley and Anderson, 2021). Therefore, and considering the irregular expression of *uvrC* observed in strain Psv-ΔgacA, we decided to construct derivatives of this strain complemented separately with genes *uvrC* (Psv::*uvrC*) and *gacA* (Psv::*gacA*) in order to perform the following phenotypical assays.

### Characterization of the GacA regulon

**Figure 2:**
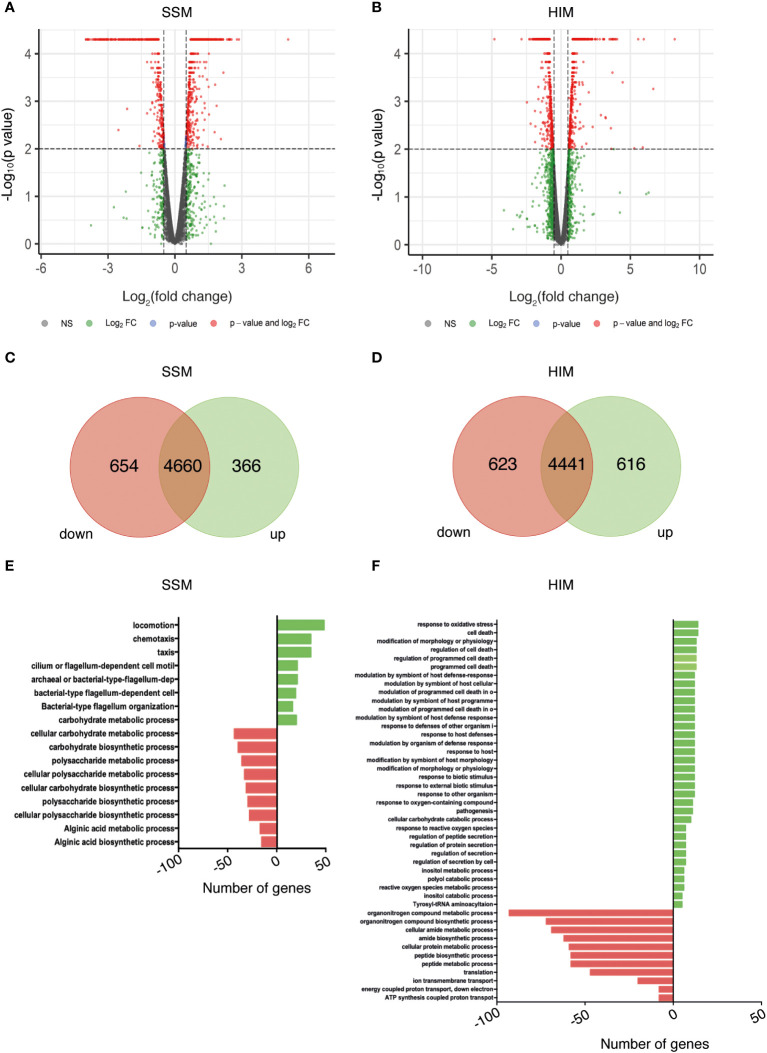
Identification of GacA-dependent genes in *P. savastanoi* pv. savastanoi NCPPB 3335 by RNA-Seq in SSM **(A, C, E)** and HIM **(B, D, F)** media. **(A, B)** Volcano plots showing DEGs in strain Psv-△gacA with respect to strain NCPPB 3335. Red dots represent significant DEGs (*q* < 0.05). **(C, D)** Venn diagrams of significant DEGs. **(E, F)** Functional categorisation of DEGs. In panels **(C**-**F)**, downregulated and upregulated DEGs are shown to the left (in red) and to the right (in green), respectively.

Distribution of the normalized FPKM values did not show significant differences between the biological replicates for any combination of strain and medium (**Supplementary Figure S2**), suggesting that no technical bias was introduced during library construction or sequencing. The normalized expression levels of wild type and mutant strains were compared to detect DEGs, which were selected considering a fold change of ± 0.5 and a statistical value of *q* < 0.05 (**Figures 2A, B**). Strain Psv-ΔgacA showed a total of 1020 (SSM) and 1239 (HIM) DEGs (**Figures 2C, D**), with 654 genes downregulated and 366 genes upregulated in SSM, and 623 genes downregulated, and 616 genes upregulated in HIM medium.

As could be expected, the growing conditions determined specific changes in transcript abundance (**Figure 2**). Thus, genes upregulated in Psv-ΔgacA in SSM medium were mainly related to bacterial motility (**Figure 2E**), but were classified in the functional categories of pathogenesis, host response and programmed cell death in HIM medium, which favours expression of virulence-related genes under the assayed conditions (**Figure 2F**). Likewise, most genes downregulated in SSM were related to the metabolism of carbohydrates (**Figure 2E**), whereas in HIM they belonged to the categories of metabolism of nitrogen and proton transport (**Figure 2F**).

In fact, there appears to be a minor overlap of the pool of genes deregulated in media SSM and HIM. On the one hand, only seven genes showed the same type of differential expression in both media (**Supplementary Figure S3**; **Supplementary Table S7**). Among these genes is *uvrC*, which is located immediately downstream of gene *gacA* and showed the highest downregulation (**Supplementary Table S7**). On the other hand, we also found opposing regulation for a limited number of genes. Specifically, 58 genes were upregulated in HIM but downregulated in SSM, while 42 genes were downregulated in HIM but upregulated in SSM (**Supplementary Figure S3**). The functions that exhibited the most notable changes in expression depending on the growth medium were related to metabolic processes or locomotion dependent on flagellum activity. These 100 genes are of considerable interest because they may represent genes that are expressed in free-living conditions (SSM) but are repressed during the interaction with the plant (HIM) and vice versa.

### Lack of GacA deregulates the expression of genes required for motility in *P. savastanoi* pv. savastanoi

**Figure 3:**
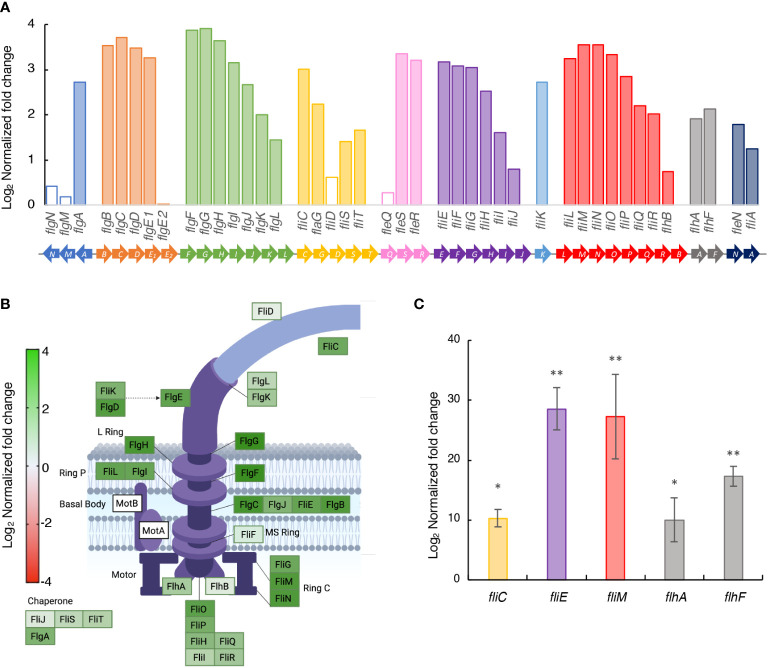
Upregulation in strain Psv-ΔgacA of genes involved in the biosynthesis of flagella in SSM medium. **(A)** Bars represents the average log_2_ of the fold change (*q* < 0.05) in the RNA-Seq analysis of strain Psv-△gacA relative to NCPPB 3335, with empty bars indicating nonsignificant different values. Genes of the same colour are part of the same operon. **(B)** Genes involved in flagellum structure. The colour gradation for each gene is proportional to the log_2_ of the fold change in strain Psv-△gacA relative to NCPPB 3335. Figure created with Biorender. **(C)** RT-qPCR analysis of selected genes upregulated in strain Psv-ΔgacA. Bars represent the average expression values of strain Psv-ΔgacA relative to those of NCPPB 3335, both of which were previously normalized to the constitutive expression of gene *gyrA*. Error bars represent the standard deviation and asterisks indicate significant differences (Student’s t-test; *p < 0.05, **p < 0.01).

Previous studies reported the involvement of the GacS/GacA system in the motility of Pto DC3000 (Chatterjee et al., 2003; Ferreiro et al., 2018; Ferreiro et al., 2021). The 42 genes for flagellar biosynthesis in diverse *Pseudomonas*, including NCPPB 3335, are grouped in nine different operons plus one monocistronic gene, *fliK* (**Figure 3A**) (McCarter, 2006). Our transcriptomic analysis in SSM medium showed that 37 out of the 42 genes were significantly upregulated in strain Psv-ΔgacA (**Figures 3A, B**). In agreement with this, genes *fliC*, *fliE*, *fliM*, *flhA* and *flhF* showed a significantly higher expression in strain Psv-ΔgacA than in strain NCPPB 3335 in an RT-qPCR analysis of cells cultivated in SSM (**Figure 3C**). This could suggest that the GacS/GacA system might directly or indirectly regulate motility in Psv by regulating flagellar biogenesis.

**Figure 4:**
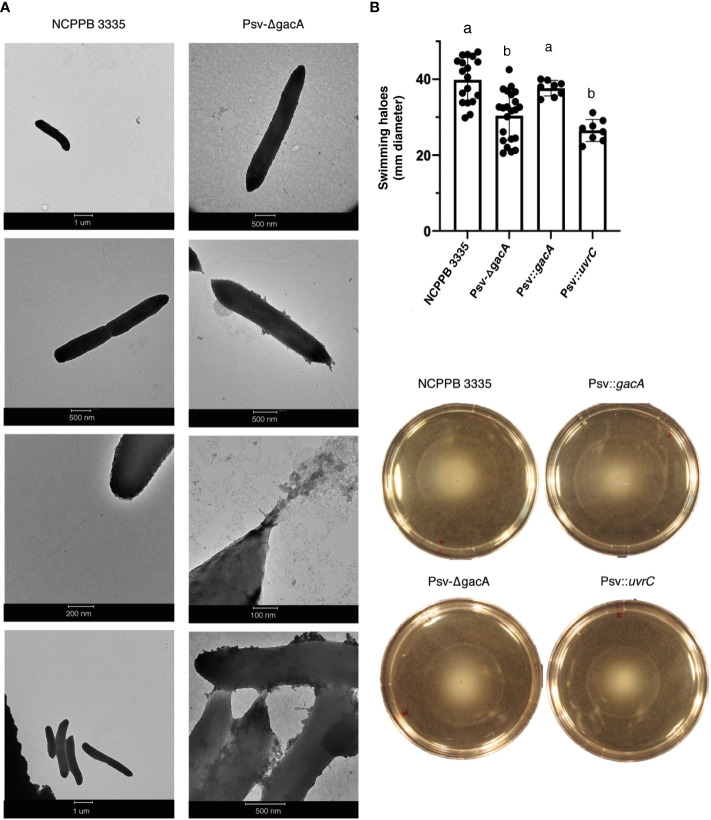
Role of GacA in motility of *P. savastanoi* pv. savastanoi NCPPB 3335. **(A)** Transmission electron microscopy (TEM) images of strains NCPPB 3335 and Psv-ΔgacA grown in SSM medium. **(B)** Swimming motility assay on LB plates. Bars indicate the average diameter of haloes, measured after 48 hours, with standard deviation, and black dots indicate the value of each repetition. Different letters indicate significant differences after Student´s t-test (*p* < 0.05). The four plates under the graph show representative plates for each of the strains, showing the swimming haloes. Psv::*gacA* and Psv::*uvrC* indicate the Psv-ΔgacA mutant complemented with genes *gacA* and *uvrC*, respectively.

Due to the significant number of deregulated genes encoding structural proteins of the bacterial flagella, we decided to analyse the morphology and motility of strain Psv-ΔgacA. Transmission electron microscopy (**Figure 4A**) showed a characteristic rod-shaped morphology with several amphitrichous flagella for the wild-type strain, whereas no regular flagella could be seen in cells from strain Psv-ΔgacA, which showed an abnormal conical morphology at their poles (**Figure 4A**).

We then tested the swimming capacity of strain Psv-ΔgacA in comparison with the wild-type strain and the *gacA* and *uvrC* complemented strains (**Figure 4B**). After 72 h, both strain Psv-ΔgacA and the complemented strain Psv::*uvrC* exhibited a significantly reduced swimming halo when compared to strain NCPPB 3335 or the complemented strain Psv::*gacA* (**Figure 4B**). These results indicate that the overexpression of flagellar genes observed in strain Psv-ΔgacA does not correlate with an increased motility or a higher abundance of flagella in the experimental conditions analysed. Additionally, the complete absence of flagella in strain Psv-ΔgacA suggest that they are not essential for swimming motility in Psv NCPPB 3335, as occurs with other bacteria (McCarren and Brahamsha, 2009; Wadhwa and Berg, 2022).

### GacA participates in the oxidative stress alleviation in Psv NCCPB 3335

Excessive levels of reactive oxygen species (ROS) or oxidants can cause cellular damage and disrupt cellular signalling processes. Therefore, a critical cell defence mechanism against oxidative stress involves the regulation of genes maintaining ROS homeostasis (Ray et al., 2012). In this sense, previous studies have shown that the GacS/GacA system enhances resistance to H_2_O_2_ and participates in the regulation of diverse genes involved in resistance to oxidative stress in biocontrol strains of *Pseudomonas* (Heeb et al., 2005; Hassan et al., 2010; Kim et al., 2014).

The RNA-Seq analysis showed a differential expression of several genes involved in the oxidative stress response, although the expression patterns were dependent on the growth medium (**Supplementary Table S8**). In cells of strain Psv-ΔgacA incubated in HIM, all the genes encoding the different subunits of the ATP synthase complex, as well as five out of 13 genes encoding the NADH oxidoreductase complex, were significantly downregulated (**Supplementary Table S8**). In contrast, 19 genes encoding catalases, peroxidases, aquaporins and reductases showed significantly higher expression levels, likely because of a differential expression of transcriptional regulator encoding genes (**Supplementary Table S8**). This pattern of regulation is essentially reversed in the Δ*gacA* mutant grown in SSM medium, where many of the genes that were downregulated in HIM medium were either upregulated or did not show a differential expression.

**Figure 5:**
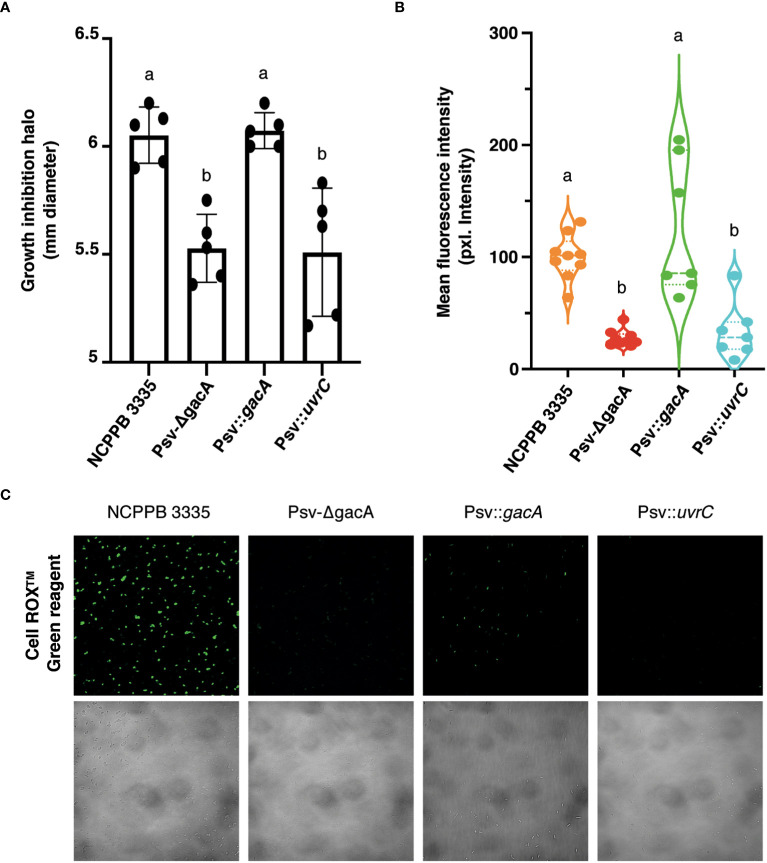
Role of GacA in tolerance to oxidative stress. **(A)** Bacterial growth sensitivity assay of the wild-type, mutant, and complemented strains to H_2_O_2_. Bars are average growth inhibition haloes with standard deviation. **(B)** Level of cell fluorescence, in intensity of pixels, in cultures after being exposed to CellROX™ Green Reagent; fluorescence is proportional to the amount of reactive oxygen species in live cells. **(C)** Representative image of confocal microscopy analysis of intrinsic oxidative stress detection in living cells using the CellROX™ Green reagent. In panels **(A, B)**, dots represent values of individual repetitions and letters indicate significant differences (Student´s t-test (*p* < 0.05). Description of strains is as in **Figure 4**.

To evaluate the role of GacA on the response of Psv to an external oxidative stress, we measured growth inhibition around a filter paper disc soaked in H_2_O_2_. After 48 hours, both strains Psv-ΔgacA and Psv::*uvrC* showed a reduced growth inhibition halo compared to strain NCPPB 3335 (**Figure 5A**). Notably, this phenotype was reversed in strain Psv::*gacA*, complemented with the *gacA* gene. Additionally, the oxidative stress state of the different mutants and the wild-type strain was measured using the cell permeable dye provided with the CellROX™ Green Reagent (Thermofisher). This dye penetrates through the plasma membrane and remains in a non-fluorescent version in a reduced state but emits fluorescence upon oxidation by ROS. The emission of green fluorescence detected by confocal microscopy was significantly reduced for both strains Psv-ΔgacA and Psv::*uvrC*, suggesting a lower abundance of ROS in their cells when compared with strains NCPPB 3335 or Psv::*gacA* (**Figures 5B, C**). These results would indicate that the activity of GacA not only contributes to a decreased tolerance to H_2_O_2_, but also to a reduction in the amount of intracellular ROS in free-living bacterial cells not exposed to oxidative stresses.

### Expression of the type III secretion system is upregulated in a Psv *gacA* mutant

**Figure 6:**
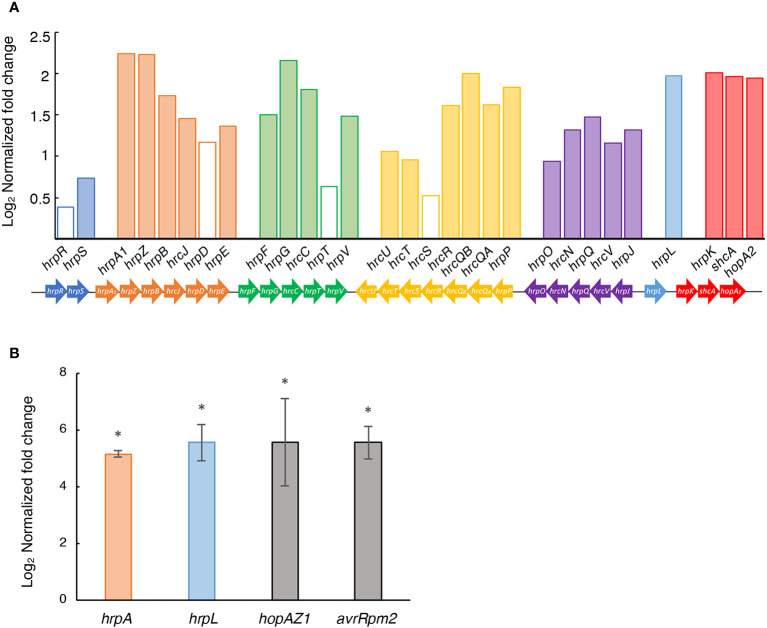
Role of GacA in the expression of the type III secretion system in HIM medium. **(A)** Bars represent the average log_2_ of the fold change (*q* < 0.05) in the RNA-Seq analysis of strain Psv-△gacA relative to NCPPB 3335, with empty bars indicating nonsignificant different values. Genes of the same colour are part of the same operon. **(B)** RT-qPCR analysis of selected genes upregulated in strain Psv-ΔgacA. Bars represent the average expression values of strain Psv-ΔgacA relative to those of NCPPB 3335, both of which were previously normalized to the constitutive expression of gene *gyrA*. Error bars represent the standard deviation and asterisks indicate significant differences (Student’s t-test; *p* < 0.05).

The T3SS is one of the most important pathogenicity factors in plant pathogenic bacteria, including Psv NCPBB 3335 (Pérez-Martínez et al., 2010), facilitating the delivery of T3Es into the host cell cytoplasm (Hueck, 1998; Göhre and Robatzek, 2008). However, the influence of the GacS/GacA pathway over the T3SS can vary among different phytopathogenic *Pseudomonas* strains (Valentini et al., 2018; O’Malley et al., 2019). Our RNA-Seq data in HIM, revealed that 25 out of the 29 genes from the *hrp*/*hrc* cluster were significantly upregulated in strain Psv-ΔgacA compared to the wild-type strain (**Figure 6A**). Additionally, nearly two thirds of the T3E genes were also upregulated in the Δ*gacA* mutant exclusively in HIM medium (**Supplementary Table S9**), suggesting that GacA also participates, directly or indirectly, in ensuring the correct level of transcription of effector genes during pathogenesis. Furthermore, RT-qPCR analyses showed that the expression levels of two T3SS regulatory genes (*hrpA* and *hrpL*) and two T3Es genes (*hopAZ1* and *avrRpm2*) were significantly increased in Psv-ΔgacA compared to the wild-type strain (**Figure 6B**).

**Figure 7:**
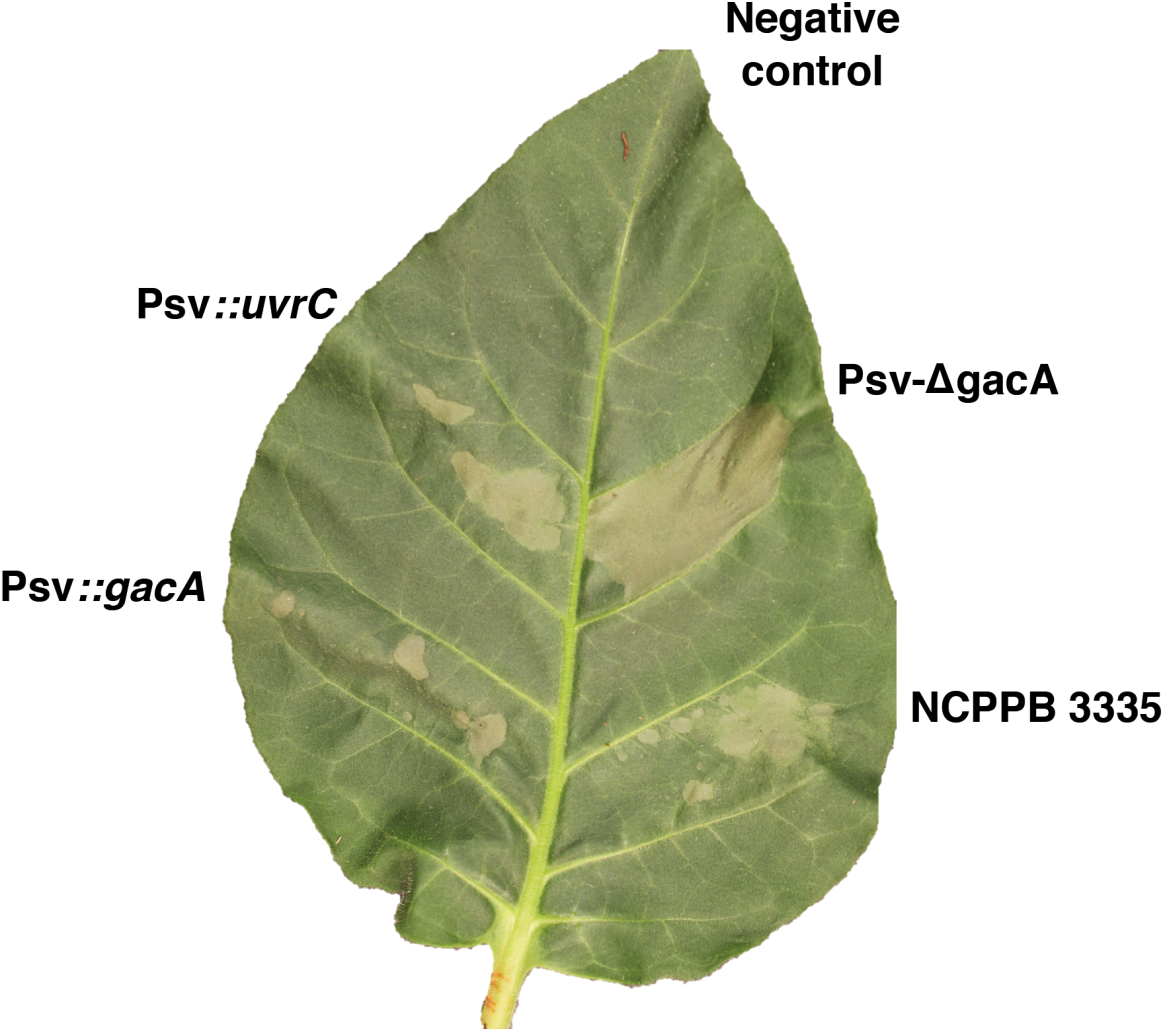
Effect of genes *gacA* and *uvrC* on the induction of the hypersensitive response (HR). *Nicotiana tabacum* var. Newdel leaves were infiltrated into the abaxial side with a total of 10^5^ CFUs of the indicated strains, and the response recorded 48 hours post-inoculation. Negative control, infiltrated leaves with 10 mM MgCl_2_; Psv::*gacA* and Psv::*uvrC* indicate the Psv-ΔgacA mutant complemented with genes *gacA* and *uvrC*, respectively.

Since genes for the biosynthesis and delivery of T3Es are overexpressed in strain Psv-ΔgacA, we evaluated its ability to induce an HR in tobacco leaves. In line with this overexpression, after 48 h of infiltration, strain Psv-ΔgacA showed a more intense HR than that observed for the wild-type strain or the *gacA* complemented strain (**Figure 7**). The Psv-ΔgacA strain complemented with gene *uvrC* consistently displayed an intermediate phenotype (**Figure 7**), which was difficult to interpret. It is possible that this is the consequence of a partial complementation in our experimental conditions, as occurs with other systems (e.g., Añorga et al., 2020; Ramírez-Zapata et al., 2020), or a possible contribution of *uvrC* to the regulation of the secretion process.

### GacA modulates virulence and fitness of Psv NCPPB 3335 in olive plants

Besides the genes presented above, several other genes involved in virulence were overexpressed in the RNA-Seq analysis of strain Psv-ΔgacA (**Supplementary Table S10**). Among these, five genes located within the *ant* and *cat* operons of the WHOP region (Caballo-Ponce et al., 2017b), a genomic island involved in the degradation of aromatic compounds, were significantly upregulated in HIM medium. Additionally, genes involved in the biosynthesis of the phytohormones indole-3-acetic acid (*iaaH1* and *iaaM1*) and cytokinins (*ptz*) were also significantly upregulated in SSM medium.

**Figure 8:**
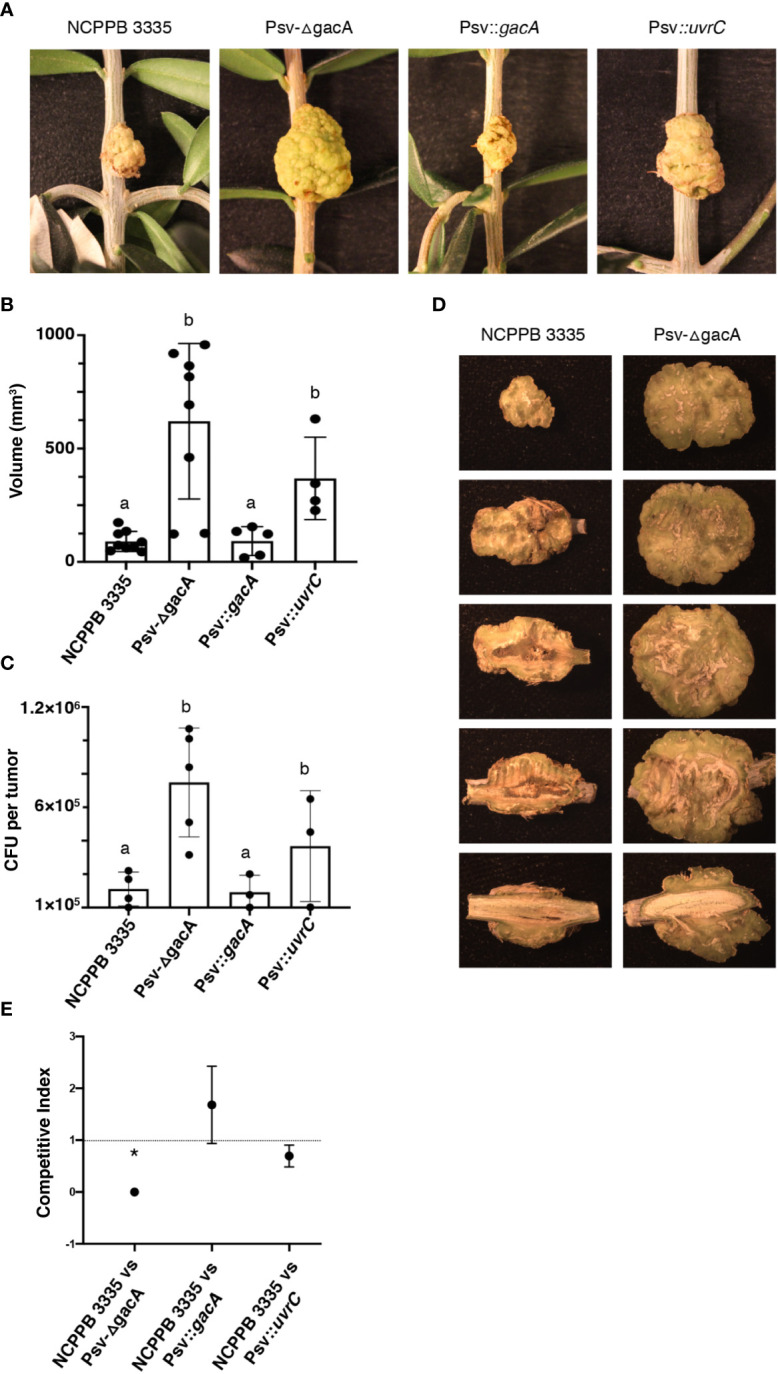
Effect of genes *gacA* and *uvrC* on the virulence of *P. savas*tanoi pv. savastanoi NCPPB 3335 in olive plants. **(A)** Symptoms generated in olive plants at 54 days post-inoculation (dpi). Negative control inoculated with 10 mM MgCl_2_. **(B, C)** Bars represent average volume of tumours **(B)** and average total bacterial populations per tumour **(C)** at 54 dpi, with dots indicating values of individual samples and error bars representing standard error. Different letters indicate means that are significantly different using ANOVA followed by Bonferroni t-test (*p* < 0.05). **(D)** Serial longitudinal sections of a representative tumour per strain; dedifferentiated tissue, apparent as greenish areas where active cell division is occurring, is predominating in tumours induced by strain Psv-ΔgacA. **(E)** Competitive index for mixed inoculations in micropropagated olive plants; points are the mean from three independent experiments with error bars indicating the standard error. The asterisk indicates a value significantly different from one using Student´s t-test with a threshold of *p* = 0.05. Description of strains is as in **Figure 4**.

To assess the possible impact on virulence of the lack of gene *gacA*, we carried out virulence assays in olive seedlings under controlled conditions. In these conditions, we measured virulence on the basis of tumour volume and total population within the tumours, as has been done previously (Rodríguez-Moreno et al., 2008; Añorga et al., 2020; Moreno-Pérez et al., 2021). After 54 dpi, tumours induced by strain Psv-ΔgacA and the complemented strain Psv::*uvrC* were noticeably bigger than tumours induced by strains NCPPB 3335 and Psv::*gacA* (**Figure 8A**). As expected, the average tumour volume was significantly higher for strain Psv-ΔgacA and its *uvrC-*complemented derivative (**Figure 8B**), while they reached significantly higher cell densities within the tumours than the wild-type and the *gacA*-complemented strains (**Figure 8C**). Nevertheless, relative bacterial populations were not significantly different for strains NCPPB 3335 and Psv-*gacA*, reaching 6.1x10^5^ and 7.4x10^5^ CFU per gram of tumour tissue, respectively. Therefore, the higher virulence of the *gacA* mutant resulted in larger tumours that, because of a higher volume, supported larger total bacterial populations. However, these tumours are not representative of typical disease symptoms because tumours induced by the wild-type strain showed a more compact and necrotized tissue, whereas those induced by strain Psv-ΔgacA had a softer and more greenish aspect and showed significant parts of the tissue occupied by aggregations of dedifferentiated cells (**Figure 8D**).

To investigate competitiveness, micropropagated olive plants were inoculated with a total of 5x10^3^ CFUs of a 1:1 ratio mixture of the wild-type strain and either 1) strain Psv-ΔgacA, 2) strain Psv-ΔgacA complemented with gene *gacA* (Psv*::gacA*), or 3) strain Psv-ΔgacA complemented with gene *uvrC* (Psv*::uvrC*). The analysis of the bacterial populations of tumours showed no significant difference in competitiveness between the wild-type strain and either one of the two complemented strains (**Figure 8E**). However, population counts of strain Psv-ΔgacA were significantly lower than those of the wild-type strain, indicating a lower competitiveness of strain Psv-ΔgacA. This indicates that genes *gacA* and *uvrC* act redundantly to increase the competitive fitness of the bacterium.

In summary, these results indicate that GacA is necessary for the development of typical disease symptoms, characterized by tumours with increased cavity formation and necrotic tissue. Conversely, the absence of this gene increases virulence, inducing significantly larger tumours with increased bacterial populations, but reduces the competitive advantage of the strain in planta.

## Discussion

The Gac-Rsm regulatory pathway has been widely studied in *Pseudomonas* (Marutani et al., 2008; Ortiz-Martín et al., 2010; Anderson et al., 2017; Zhang et al., 2018; Ramírez-Zapata et al., 2020; Zhang et al., 2020). GacA transcriptionally targets small *rsm* RNAs, which control the activity of post-transcriptional regulatory proteins of the Csr/Rsm family (Sobrero and Valverde, 2020). Species of *Pseudomonas* contain a variable number of *rsm* sRNAs, typically seven in the analysed strains of *P. syringae sensu lato*, and from two to seven functional *csr/rsm* protein genes (Sobrero and Valverde, 2020). In this work, we identified seven *rsm* sRNAs and four different Rsm/Csr gene homologues (*rsmA*, *rsmC*, *rsmE* and *rsmH1*) in the genome of Psv NCPPB 3335 (**Table 1**). RsmA and RsmE from Psv NCPPB 3335 showed the highest identity (100 %) with their respective homologues in Pto DC3000 and Pph 1448A (**Table 1**), suggesting a highly conserved and relevant function for these two proteins in bacteria belonging to *P. syringae sensu lato*. However, despite the high conservation of all these molecules, their regulatory roles are strain dependent, as has been shown for the regulation of the T3SS in *P. syringae* (Chatterjee et al., 2003; Marutani et al., 2008; O’Malley et al., 2020; Ramírez-Zapata et al., 2020; O’Malley and Anderson, 2021); and the production of iron-chelating siderophores in *P. fluorescens* (Cheng et al., 2013; Yu et al., 2014). These regulatory differences might reflect differential affinities of sRNAs to different RsmA homologues and/or distinct expression patterns (Sonnleitner and Haas, 2011; Ge et al., 2019). In fact, in Pph 1448A, GacA regulates the expression of six of the seven sRNAs but does not alter expression of genes encoding Rsm proteins (Ramírez-Zapata et al., 2020). However, in Psv NCPPB 3335, GacA modulated the expression of RsmE and RsmH1 and two sRNAs under the tested conditions (**Table 1**).

In many bacterial species, the *gacA* gene is recurrently located upstream of the *uvrC* gene (Goodier and Ahmer, 2001; Heeb and Haas, 2001), coding for subunit UvrC of the nucleotide excision repair endonuclease UvrABC, which catalyses removal of very diverse chemical and structural DNA lesions. Additionally, *uvrY* (the *gacA* homologue in *E. coli*) and *uvrC* appear to form a single transcriptional unit in *E. coli* K12 (Moolenaar et al., 1987), whereas *gacA* mutants of *P. fluorescens*, *P. aeruginosa*, *Salmonella typhimurium* and *Erwinia carotovora* showed a significant UV tolerance reduction, suggesting that both genes could also be co-transcribed in these strains (Laville et al., 1992; Reimmann et al., 1997; Ahmer et al., 1998; Eriksson et al., 1998). A 5´ RACE analysis revealed that the 5´-UTR of the *uvrC* transcripts in the wild-type strain, Psv NCPPB 3335, was located within the *gacA* CDS (**Figures 1B**, **Supplementary Figure S1A**). Therefore, it was unexpected that the RNA-Seq data for strain Psv-ΔgacA showed the existence of a minor amount of complete *uvrC* transcripts as part of the total RNA samples (**Supplementary Figure S1B**). These transcripts likely originate from a functional promoter preceding gene *gacA*. Additionally, RT-qPCR analyses showed a significantly reduced, but still detectable, transcription of *uvrC* in strain Psv-ΔgacA (**Figure 1A**). These results suggest that *gacA* and *uvrC* form a co-transcriptional unit in Psv NCPPB 3335, as occurs in other bacteria, but that *uvrC* can still be transcribed from an alternative promoter located within the *gacA* CDS. This situation also likely occurs in many other bacteria. For instance, *uvrC* can be transcribed from three functional promoters in *E. coli*, although only the distal one, located more than 1 kb upstream of the *uvrC* start codon and likely preceding *gacA*, leads to an efficient synthesis of the UvrC product (Sharma et al., 1984). Additionally, polar *gacA* mutations in Pto DC3000 and Pph 1448A reduced, but did not abolish, transcription of *uvrC* (O’Malley et al., 2019; Ramírez-Zapata et al., 2020). Nevertheless, reduction of the *uvrC* expression due to mutations on *gacA* might confound the assessment of the GacA regulon because of the demonstrated changes in diverse phenotypes and gene expression patterns in *uvrC* mutant backgrounds in other bacteria (Humann et al., 2009; O’Malley et al., 2019). It is unclear how the lack of UvrC could determine widespread transcriptional changes, although it may be due to reductions in DNA repair activities or a failure to adequately respond to cellular stresses (Humann et al., 2009; O’Malley et al., 2019). However, we are confident in our identification of phenotypes regulated by GacA because we routinely used as controls derivative strains of Psv-ΔgacA complemented with either the *gacA* or the *uvrC* gene.

Many phytopathogenic bacteria combine epiphytic and endophytic lifestyles during their host colonization process, for which bacterial motility is crucial to enable colonization of different niches by microorganisms. For instance, flagella formation, as well as swimming, swarming, and twitching movements, are negatively regulated by GacS/GacA in the biocontrol strains *P. fluorescens* F113 (Navazo et al., 2009) and *P. chlororaphis* O6 (Kim et al., 2014). *P. fluorescens* strains carrying a *gacS* deletion produce higher swimming haloes (Martínez-Granero et al., 2006), however, this phenotype disappears when RsmA and RsmE are overexpressed in this mutant (Martínez-Granero et al., 2012). Additionally, GacA is required by Pto DC3000 for swarming and swimming motility (Chatterjee et al., 2003; Vargas et al., 2013), as well as for full virulence on leaf surfaces (O’Malley et al., 2020). In this sense, our RNA-Seq analysis showed that 37 out of 42 genes encoding the flagella machinery were significantly upregulated in strain Psv-ΔgacA grown in SSM (**Figure 3**), but not in HIM medium. However, in spite of the higher expression of flagella genes, Psv-ΔgacA showed smaller swimming haloes than the wild-type strain NCPPB 3335 or the Psv::*gacA* complemented strains (**Figure 4B**). Intriguingly, the mutant strain Psv-ΔgacA was unable to polymerize regular flagella, showing an abnormal cone-shaped morphology at the cell poles (**Figure 4A**). Since swimming motility depends on a correct flagella disposition, the reduced motility haloes (**Figure 4B**) would likely be caused by the inability of strain Psv-ΔgacA to assemble regular flagella (**Figure 4A**). Reduced swimming capacities and altered colony morphologies have also been reported in GacA mutants of *P. aeruginosa* and *P. fluorescens* (Goodier and Ahmer, 2001). Furthermore, similar phenotypes have also been observed in animal pathogenic bacteria carrying mutations in gene *csrA*, i.e., *Legionella pneumophila* (Molofsky and Swanson, 2003), *Bacillus subtilis* (Yakhnin et al., 2007) or *Borrelia burgdorferi* (Sanjuan et al., 2009). All these results together demonstrate a connection between the Gac-Rsm regulatory pathway and bacterial motility and morphology in gamma proteobacteria (Goodier and Ahmer, 2001; O’Malley and Anderson, 2021; Song et al., 2023). Nevertheless, it remains to be elucidated whether the effect of *gacA* in the expression of flagellar genes takes place at the transcriptional or post-transcriptional levels. For instance, LetA, the GacA homologue of *L. pneumophila*, regulates expression of flagellar genes in an sRNA-independent manner (Sahr et al., 2009). Proteomics analyses would likely contribute to address this issue in Psv.

One of the earliest plant defence mechanisms is the generation of reactive oxygen species (ROS), contributing, together with the acidic pH in the apoplast, to cause oxidative stress (Sharma et al., 2012). In turn, GacS/GacA participates in the regulation of genes involved in the metabolism of ROS. For instance, ROS-related genes are upregulated in GacS/GacA mutants of various *P. fluorescens* and *P. protegens* strains (Whistler et al., 1998; Hassan et al., 2010; Cheng et al., 2013). In *P. fluorescens* SBW25, the *sod* gene cluster, including a super-oxide dismutase (*sodA*) and a fumarate hydratase (*fumC1*), was among the most differentially regulated locus in a *gacS* mutant (Cheng et al., 2013). Likewise, diverse genes related to the redox status of the cell also appear to be regulated by the GacS/GacA system in Psv NCPPB 3335, depending strongly on the growth medium (**Supplementary Table S8**). In particular, the *soxR* gene, which encodes a negative LysR family transcriptional regulator with a critical role in oxidative stress responses in numerous bacteria (Flores-Cruz and Allen, 2011; Toledo et al., 2011; Burbank and Roper, 2014), was inversely deregulated in Psv-ΔgacA during growth in SSM (upregulated) and HIM (downregulated) (**Supplementary Table S8**). Therefore, this suggests that GacA could indirectly regulate the expression of genes related to the oxidative stress response through the transcriptional regulatory activity of SoxR. Genes encoding the NADH-quinone oxidoreductase complex also show a similar inverse deregulation in SSM and HIM media (**Supplementary Table S8**). Considering that HIM media simulates the oxidative environment found for bacterial cells during plant invasion, a reduced activity of the NADH-quinone oxidoreductase complex could avoid the generation of additional ROS within the bacterial cells. Nevertheless, we cannot establish a clear relationship between gene expression mediated by GacA in NCPPB 3335 and its resistance to oxidative stresses. Thus, GacA mediates repression of various catalases, peroxidases, or reductases genes in HIM medium (**Supplementary Table S8**), although we would have expected that these genes would be highly expressed natively because HIM mimics the oxidative conditions found in the plant apoplast. Additionally, we identified only a few genes potentially involved in resistance to oxidative stresses that were upregulated in Psv-ΔgacA, even though this strain showed a higher tolerance to hydrogen peroxide in *in vitro* assays (**Figure 5A**) and a higher capacity to reduce the accumulation of ROS inside the bacterial cells than the wild-type strain (**Figures 5B, C**). Finally, we did not observe a significant deregulation of other genes that are involved in tolerance and detoxification of ROS, such as the *sod* locus or genes *pqiB* and *rubB*, previously identified in Psv NCPPB 3335 as required for survival in olive plant tissues (Matas et al., 2012). Therefore, in Psv NCPPB 3335 GacA participates, directly or indirectly, in the regulation of genes involved in maintaining the redox status of the cell and the tolerance to oxidative stresses, but further research is necessary to identify the genes involved in these functions and the regulatory level at which GacA exerts its action.

Induction of T3SS genes in *P. syringae sensu lato* cultures requires incubation in the synthetic HIM medium used in this study (Huynh et al., 1989), which mimics apoplast conditions. Incubation of low cell density cultures in this medium allowed the evaluation of the role of the Gac-Rsm system in *P. syringae* pathogens of bean, tomato, and tobacco plants (Chatterjee et al., 2003; Marutani et al., 2008; Ortiz-Martín et al., 2010; Ferreiro et al., 2018). The role of GacS/GacA in regulation of the T3SS, one of the most relevant pathogenicity factors in phytopathogenic bacteria (Alfano and Collmer, 2004), is variable among *P. syringae* strains (Hirano et al., 1997; Chatterjee et al., 2003; Marutani et al., 2008; Ortiz-Martín et al., 2010; Vargas et al., 2013). GacA negatively regulates T3SS genes in Pto DC3000 and Pph 1448A (Chatterjee et al., 2003; Ortiz-Martín et al., 2010). Similarly, GacA negatively regulates both the T3SS and effector genes in NCPPB 3335 (**Figure 6**, **Supplementary Table S9**) and the strain with a mutated *gacA* gene produced a more intense and expanded HR reaction in tobacco (**Figure 7**), unlike in strains DC3000 and 1448A. Remarkably, overexpression of the T3SS and T3Es in Psv-ΔgacA also resulted in increased virulence, characterized by significantly larger tumour volume and bacterial populations for inoculations of strain Psv-ΔgacA. However, the tumours showed an immature appearance and a greenish colour, with no apparent tissue necrosis, indicating an altered infection process (**Figures 8A, C**). We do not have a satisfactory explanation for the larger tumours produced by the *gacA* mutant, because genes involved in the biosynthesis of phytohormones are not significantly differentially expressed in medium HIM (**Supplementary Table S10**). It is possible, however, that the overexpression of gene *idi* (**Supplementary Table S10**) could contribute to an imbalance of phytohormones impacting the development of tumours, as shown before with mutants in this gene (Añorga et al., 2020). Despite the higher population of cells reached in planta by Psv-ΔgacA, this strain showed a significantly reduced competitiveness. Therefore, our assays suggest that the ability of the *gacA* mutant to grow in the plant tissue is apparently not impaired when infecting the plant by itself. However, the infection process is likely affected because it cannot colonize the tissue as efficiently as the wild-type strain. These results underscore the variability of the GacA regulon in diverse strains, as a *gacA* mutant of Pto DC3000 was not affected in virulence in *Arabidopsis* whereas that of Pph 1448A showed a moderately reduced virulence on bean (*Phaseolus vulgaris*).

In summary, in this work we analysed the GacA regulon of Psv using an RNA-Seq analysis in a standard minimal growth medium, SSM, and a *hrp*-inducing medium, HIM. We identified DEGs in both growth media, suggesting differences in the regulation of metabolic pathways depending on environmental conditions. Most of the genes significantly upregulated in Psv-ΔgacA in SSM medium were related to bacterial motility. Conversely, the most relevant genes significantly upregulated in HIM medium were those involved in the redox balance and tolerance to oxidative stress, as well as those encoding the T3SS and T3Es. The phenotypic analyses shown here demonstrate that GacA participates, directly or indirectly, in the regulation of relevant physiological processes in Psv NCPPB 3335, including motility, resistance to oxidative stress, virulence, and fitness during invasion and colonization of plant tissue. Further proteomics analysis would be required to determine the level at which GacA exerts its function in this bacterial pathogen of woody hosts.

## Data availability statement

The datasets presented in this study can be found online at the NCBI, accession number GSE254022.

## Author contributions

CL-B: Data curation, Formal analysis, Investigation, Methodology, Software, Writing – original draft. JM: Conceptualization, Data curation, Formal analysis, Methodology, Supervision, Writing – original draft, Writing – review & editing, Funding acquisition. MM-G: Investigation, Methodology, Writing – original draft. CR: Conceptualization, Data curation, Formal analysis, Funding acquisition, Methodology, Project administration, Supervision, Writing – original draft. Writing – review & editing. LR-M: Conceptualization, Data curation, Formal analysis, Funding acquisition, Methodology, Project administration, Supervision, Writing – original draft, Writing – review & editing.
