# Supplementary material for: GacA reduces virulence and increases competitiveness in planta in the tumorigenic olive pathogen Pseudomonas savastanoi pv. savastanoi
Source: Front Plant Sci. 2024 Feb 5;15:1347982. doi: 10.3389/fpls.2024.1347982 (PMC10875052; doi:10.3389/fpls.2024.1347982)
Supplement: Supplementary file 1 [file DataSheet_1.pdf]

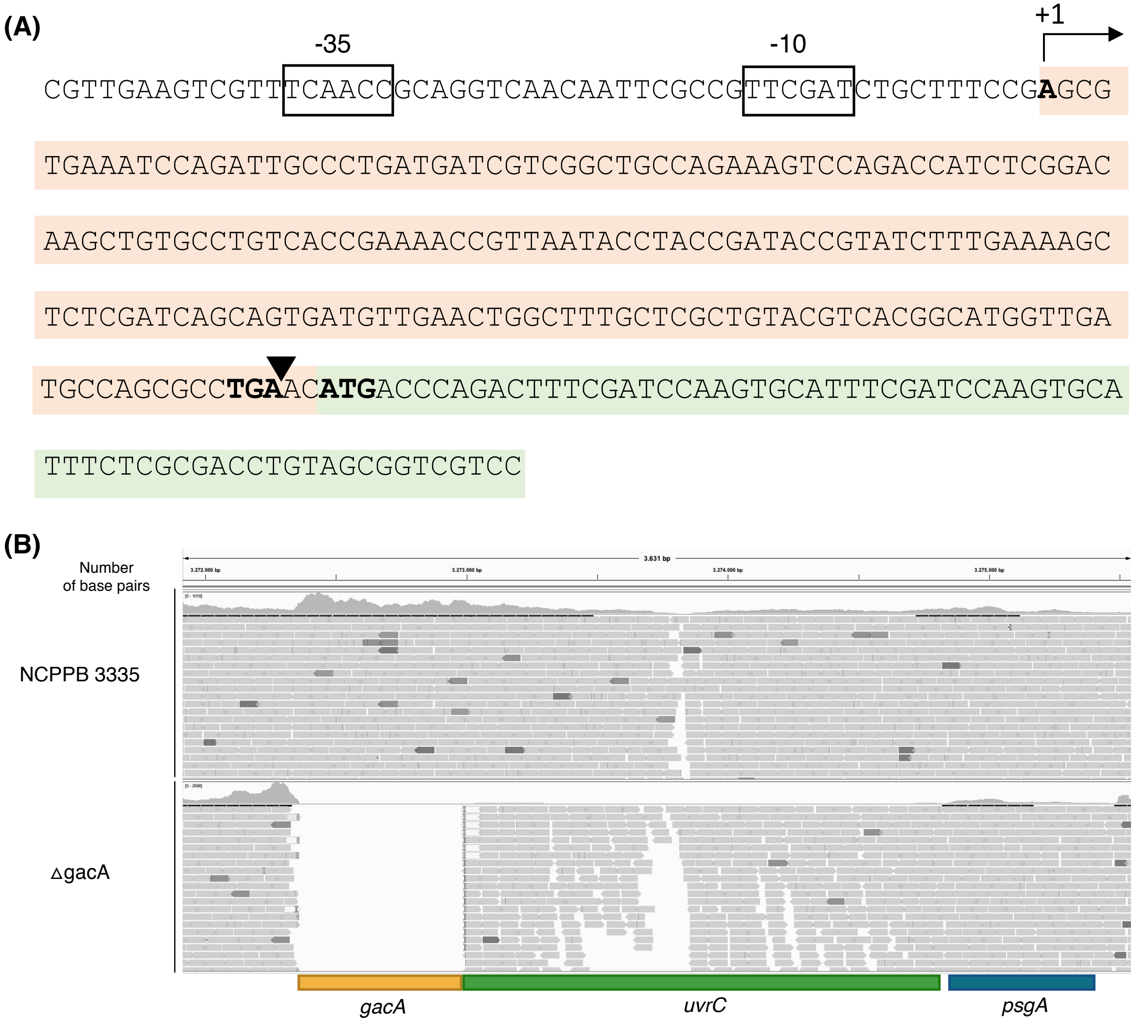


**Figure S1.** Characterization of genes *gacA* and *uvrC* genomic context of *Pseudomonas savas*tanoi pv. savastanoi NCPPB 3335. **(A)** The predicted promoter regions (-35 and -10) of the *uvrC* gene, in accordance with a 5’ RACE assay, are boxed. The predicted 5' untranslated region (UTR) of the *uvrC* gene and the CDS are filled in light red and green, respectively, with the transcription start site (+1) and the predicted start codon in boldface. The deletion of gene *gacA* covers a fragment starting immediately before the start codon (not shown in this figure) to the stop codon, shown in bold, and with the 3’ end indicated with a vertical black arrowhead. **(B)** Representation of the number of reads for the *gacA-uvrC-psgA* gene region by RNA-Seq on HIM medium. The upper section shows the read counts obtained in this region in the wild-type strain, while the lower section represents the read counts in strain Psv-∆gacA.
