# Supplementary material for: GacA reduces virulence and increases competitiveness in planta in the tumorigenic olive pathogen Pseudomonas savastanoi pv. savastanoi
Source: Front Plant Sci. 2024 Feb 5;15:1347982. doi: 10.3389/fpls.2024.1347982 (PMC10875052; doi:10.3389/fpls.2024.1347982)
Supplement: Supplementary file 2 [file DataSheet_2.pdf]

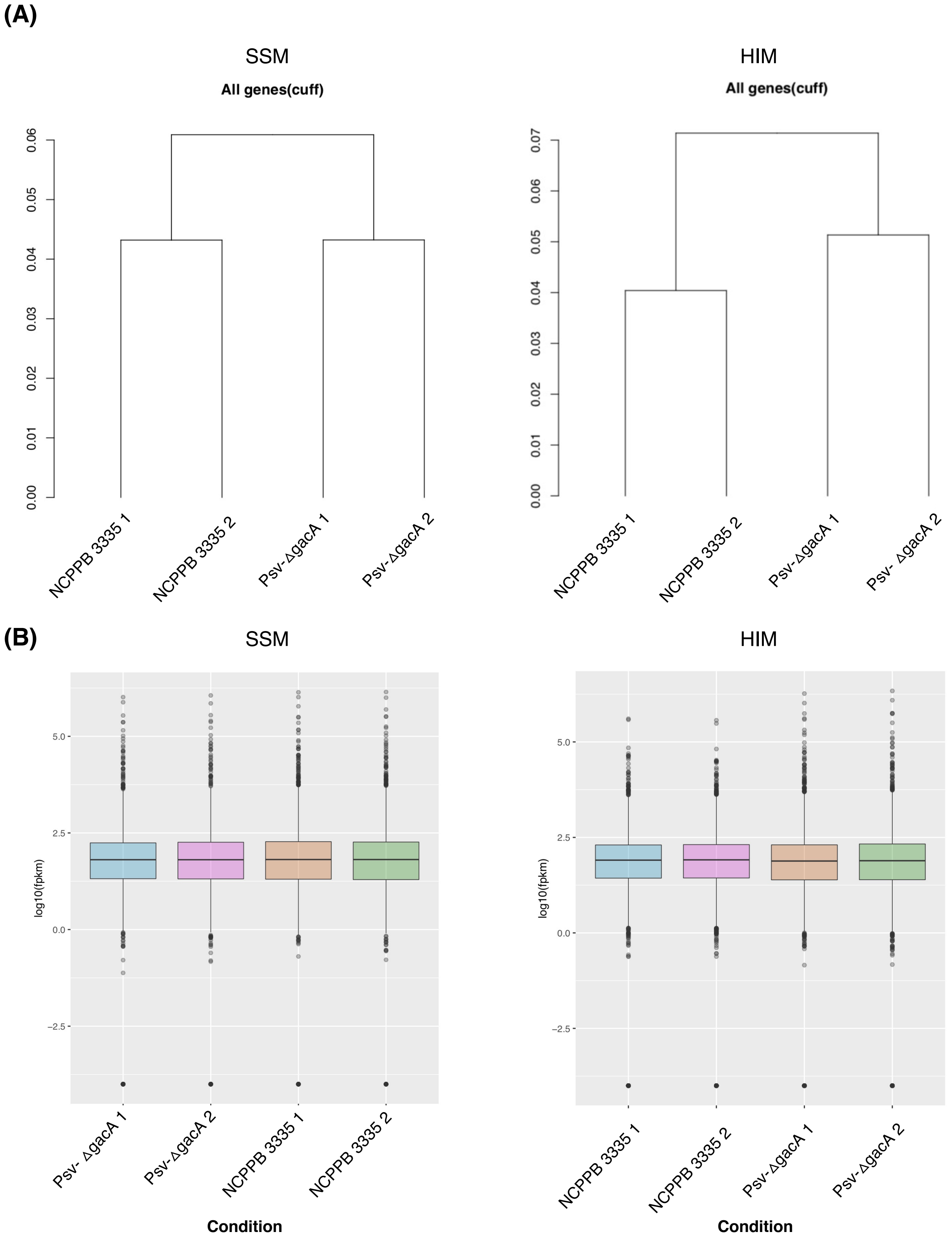


**Figure S2.** Identification of GacA-dependent genes in *Pseudomonas savastanoi* pv. savastanoi NCPPB 3335 in SSM and in HIM media by RNA-Seq analysis. To characterize the GacA regulon, clean reads of each replicate were compared using the cummeRbund R package (Goff et al., 2012). **(A)** Pooling of the NCPPB 3335 and Psv-ΔgacA samples used in RNA-Seq analysis in SSM (left) and in HIM (right) media. **(B)** Analysis of the gene expression of the biological replicates of the NCPPB 3335 strain and the Psv-ΔgacA strain used in the RNA-Seq analysis. The box plot represents the distribution of the normalized gene expression values of the biological replicates of both strains in SSM medium (left) and in HIM medium (right).
