# Supplementary material for: GacA reduces virulence and increases competitiveness in planta in the tumorigenic olive pathogen Pseudomonas savastanoi pv. savastanoi
Source: Front Plant Sci. 2024 Feb 5;15:1347982. doi: 10.3389/fpls.2024.1347982 (PMC10875052; doi:10.3389/fpls.2024.1347982)
Supplement: Supplementary file 3 [file DataSheet_3.pdf]

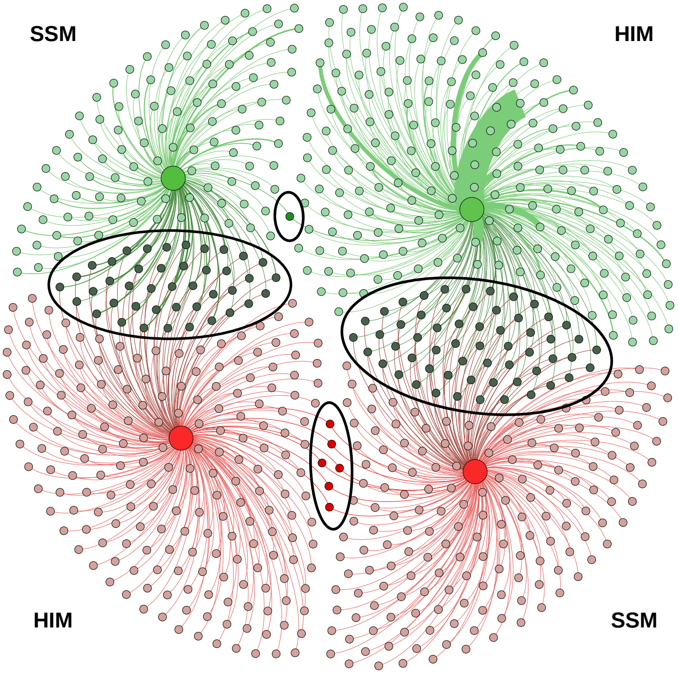

Figure S3. Identification of genes from *Pseudomonas savastanoi* pv. savastanoi NCPPB 3335 whose regulation by GacA changes with the type of culture medium. The dot plot shows the total number of genes regulated by GacA in media SSM and HIM, with each dot representing a gene. The upper half (green) and the lower half (red) represent, respectively, upregulated, or downregulated genes in strain Psv-△gacA in media SSM and HIM. Within the vertical ovals the number of genes that change in regulation depending on the medium in which the culture is grown is indicated.
