# Supplementary material for: GacA reduces virulence and increases competitiveness in planta in the tumorigenic olive pathogen Pseudomonas savastanoi pv. savastanoi
Source: Front Plant Sci. 2024 Feb 5;15:1347982. doi: 10.3389/fpls.2024.1347982 (PMC10875052; doi:10.3389/fpls.2024.1347982)
Supplement: Supplementary file 4 [file DataSheet_4.pdf]

Table S1. Strains used in this study.

Strain	Main features	Reference or source
Escherichia coli		
DH5α	*F -, ϕ80dlacZ M15, (lacZYA-argF) U169, deoR, recA1, endA, hsdR17 (rk - mk -), phoA, supE44, thi-1, gyrA96, relA1*	Hanahan, 1983
GM2929	*F -, ara-14, leuB6, thi-1, tonA31, lacY1, tsx-78, galK2, galT22, glnV44, hisG4, rpsL136, xyl-5, mtl-1, dam13: Tn9, dcm-6, mcrB1, hsdR2, mcrA, recF143 (*Sp^R, Cm^R)	Bullock et al., 1987
***Pseudomonas savastanoi* pv. savastanoi**		
NCPPB 3335	Wild-type strain isolated from olive	Pérez-Martínez et al., 2007
Psv-ΔgacA Km^R	Derived from Psv NCPPB 3335, the complete *gacA* gene was deleted and replaced with a Km-resistance gene (Km^R)	This work
Psv-ΔgacA	*gacA* mutant derived from Psv-ΔgacA Km^R by removal of the Km^R gene	This work
Psv*::gacA*	*gacA* mutant derived from Psv-ΔgacA complemented with with gene *gacA* using pMMG (Km^R)	This work
Psv*::uvrC*	*gacA* mutant derived from Psv-ΔgacA complemented with gene *uvrC* using pBBR:*uvrC* (Gm^R)	This work
