# Supplementary material for: GacA reduces virulence and increases competitiveness in planta in the tumorigenic olive pathogen Pseudomonas savastanoi pv. savastanoi
Source: Front Plant Sci. 2024 Feb 5;15:1347982. doi: 10.3389/fpls.2024.1347982 (PMC10875052; doi:10.3389/fpls.2024.1347982)
Supplement: Supplementary file 5 [file DataSheet_5.pdf]

Table S2. Plasmids used in this study.

Name	Description	Reference
pGEM-T	Cloning vector containing *ori* f1 and *lacZ* (Ap^R)	(Promega, USA)
p*gacA*-Km	pGEM-T derivate, contains approximately 1.2 kb on each side of the *P. savastanoi* pv. savastanoi NCPPB 3335 *gacA* gene (Ap^R, Km^R)	This work
pMMG-Km	pBBR1MCS-5 derivative, contains *P. savastanoi* pv. savastanoi NCPPB 3335 open reading frame of the *gacA* gene and its promoter region flanked by EcoRI-BamHI restriction sites (Gm^R)	This work
pBBR:*uvrC*	pBBR1MCS-2 derivative, contains the complete coding sequence of gene *uvrC* and its promoter region from *P. savastanoi* pv. savastanoi NCPPB 3335 flanked by XhoI-EcoRV restriction sites (Km^R)	This work
pFLP2	Contains an Flp recombinase gene and *sacB* (Ap^R)	Hoang et al., 1998
