# Supplementary material for: GacA reduces virulence and increases competitiveness in planta in the tumorigenic olive pathogen Pseudomonas savastanoi pv. savastanoi
Source: Front Plant Sci. 2024 Feb 5;15:1347982. doi: 10.3389/fpls.2024.1347982 (PMC10875052; doi:10.3389/fpls.2024.1347982)
Supplement: Supplementary file 6 [file DataSheet_6.pdf]

Table S3. List and application of primers used in this work.

Purpose	Name	Sequence (5´→ 3´)
Mutagenize *gacA*	*gacA*-FA-F	GAGCATTGACGTGGTGGTCCAG
	gacA-FA-R	CCCTATAGTGAGTCGGATCCGCAGACACCTCGCGCAACGG
	gacA-FD-F	GGATCCGACTCACTATAGGGACATGACCCAGACTTTCGATCC
	gacA-FD-R	CAATCAACGCCGGGAAGTC
Complement *gacA*	*gacA*-F	AAAAGAATTCGTTGGGCAAAGATGATCAG
	gacA-R	AAAAGGATCCGCACTTGGATCGAAAGTC
Complement *uvrC*	*uvrC*-F	AAAACTCGAGCTCGATCAGCAGTGATGTTG
	uvrC-R	AAAACTATAGCTACGAGGTGAGCGGGCATT
Real time quantitative PCR	*gyrA*-F	TTCCAGTCGTTACCCAGCTCG
	gyrA-R	GACGAGCTGAAGCAGTCCTACC
	fliM-F	GCTCTCCCAGGATGAGATCG
	fliM-R	GACTGGTCAGGTCGTAGCTT
	fliC-F	ACCTCGATGACTCGTCTGTC
	fliC-R	GATCGCCATTGTCTGACCAC
	fliE-F	GTTGGATATGCGGGCCATG
	fliE-R	CCGCTCTTGCCGATTTCAAA
	flhA-F	CGTACACATCCGTGACAACC
	flhA-R	GCTCGCGATCCGGATAAATC
	flhH-F	CTATGATTCGATGCGCTCCG
	flhH-R	CTCGTCAATCTCCGGGATCA
	hrpA-F	GTGTCGTTAACACAGTGG
	hrpA-R	ACTGGACGACCGAGTTCC
	hrpL-F	CCTAGTGATCCTTGATGC
	hrpL-R	CAAGCAATCAATGGCTGC
	hopAZ1-F	CAACAACAATTGGGGCCTCA
	hopAZ1-R	TCGATAGTGGCGATGCAGAT
	avrRpm2-F	TTCTGGCTCCCATCATGTGT
	avrRpm2-R	CGTGCCTCTTCGGAAAGATG
	psgA-F	AAATGCTCGCGCTGGTTATT
	psgA-R	TTTCTGCCGTAGTCTTGAGGT
5`RACE	SP1	CCGATGAATACTCAATCGCGGGT
	SP2	CCGTGATGGTCGTTTCAATCTGG
	SP3	GAAAATAGCTGGCAAGACGCTTC
