# Supplementary material for: GacA reduces virulence and increases competitiveness in planta in the tumorigenic olive pathogen Pseudomonas savastanoi pv. savastanoi
Source: Front Plant Sci. 2024 Feb 5;15:1347982. doi: 10.3389/fpls.2024.1347982 (PMC10875052; doi:10.3389/fpls.2024.1347982)
Supplement: Supplementary file 7 [file DataSheet_7.pdf]

Table S4. Readings obtained from the RNA-Seq analysis in SSM medium of *Pseudomonas savastanoi* pv. savastanoi NCPPB 3335 and its Δ*gacA* mutant.

Sample	Raw reads	Clean reads	Unmapped reads (%)[a]	Reads mapped to reference (%)[b]
		**NCPPB 3335**		
Replicate 1	37,725,357	36,194,592	2.4	99.9
Replicate 2	37,246,194	35,130,621	4.1	99.1
Total	74,971,551	71,325,213	4.9	99.5
		**Psv-ΔgacA**		
Replicate 1	36,221,434	34,841,624	2.3	99.6
Replicate 2	38,553,120	36,998,627	2.5	99.5
Total	74,774,554	71,840,251	3.9	99.5
**Total RNA-seq**	149,746,105	143,165,464	4.4	99.5

[a] Percentage of rejected reads obtained after pre-processing with SeqTrimNext.

[b] Percentage of clean reads that aligned with the reference genome: the concatenated sequence of the chromosome and the three native plasmids (pPsv48A, pPsv48B and pPsv48C) of strain Psv NCPPB 3335.
