# Supplementary material for: GacA reduces virulence and increases competitiveness in planta in the tumorigenic olive pathogen Pseudomonas savastanoi pv. savastanoi
Source: Front Plant Sci. 2024 Feb 5;15:1347982. doi: 10.3389/fpls.2024.1347982 (PMC10875052; doi:10.3389/fpls.2024.1347982)
Supplement: Supplementary file 8 [file DataSheet_8.pdf]

Table S5. Readings obtained in the RNA-Seq analysis in HIM medium of *Pseudomonas savastanoi* pv. savastanoi NCPPB 3335 and its Δ*gacA* mutant.

Sample	Raw reads	Clean reads	Unmapped reads (%)[a]	Reads mapped to reference (%)[b]
		**NCPPB 3335**		
Replicate 1	41,289,538	33,083,850	19.9	99.8
Replicate 2	40,704,762	33,925,884	16.7	99.6
Total	81,994,300	67,009,734	18.3	99.7
		**Psv-ΔgacA**		
Replicate 1	33,148,617	31,728,474	2.7	99.6
Replicate 2	33,718,416	32,324,226	2.5	99.6
Total	66,867,033	64,052,700	4.2	99.6
**Total RNA-seq**	148,861,333	131,062,434	12	99.6

[a] Percentage of rejected reads obtained after pre-processing with SeqTrimNext.

[b] Percentage of clean reads that aligned with the reference genome: the concatenated sequence of the chromosome and the three native plasmids (pPsv48A, pPsv48B and pPsv48C) of strain Psv NCPPB 3335
