# Supplementary material for: GacA reduces virulence and increases competitiveness in planta in the tumorigenic olive pathogen Pseudomonas savastanoi pv. savastanoi
Source: Front Plant Sci. 2024 Feb 5;15:1347982. doi: 10.3389/fpls.2024.1347982 (PMC10875052; doi:10.3389/fpls.2024.1347982)
Supplement: Supplementary file 9 [file Table_6.pdf]

Table S6. Percentage of gene coverage of reads from the different samples subjected to RNA-Seq analysis[a].

	SSM				HIM			
	NCPPB 3335		Psv-ΔgacA		NCPPB 3335		Psv-ΔgacA	
**Characteristics**	**Replicate 1**	**Replicate 2**	**Replicate 1**	**Replicate 2**	**Replicate 1**	**Replicate 2**	**Replicate 1**	**Replicate 2**
Sequencing depth[b]	441.80	428.90	425.40	451.70	344.20	351.60	387.40	394.60
Chromosomal coverage[c]	99.40	99.40	99.35	99.35	99.44	99.44	99.44	99.44
Plasmid coverage[c]	100.00	100.00	100.00	100.00	100.00	100.00	100.00	100.00

[a] Reads were obtained by Illumina sequencing (75 nt of read length); in all cases more than 88 % of reads had a Phred score ≥Q30.

[b] Sequencing depth was defined as number of nucleotides used for the analysis divided by the total genome size (chromosome plus the three plasmids), which amounts to 6,143,577 nt for the wild-type strain and to an estimate of 6,142,932 for strain Psv-ΔgacA.

[c] Gene coverage was calculated over the 5680 genes annotated in the GenBank records of the chromosome (accession no. CP008742.1) and the three plasmids, with accession numbers pPsv48A: FR820585; pPsv48B: FR820586 and pPsv48C: FR820587
