# Supplementary material for: GacA reduces virulence and increases competitiveness in planta in the tumorigenic olive pathogen Pseudomonas savastanoi pv. savastanoi
Source: Front Plant Sci. 2024 Feb 5;15:1347982. doi: 10.3389/fpls.2024.1347982 (PMC10875052; doi:10.3389/fpls.2024.1347982)
Supplement: Supplementary file 10 [file Table_7.pdf]

Table S7. The seven genes that in strain Psv-ΔgacA have the same differential regulation in media SSM and HIM, identified by RNA-Seq.

Locus tag[a]	Annotation	FPKM[b]				Fold change[c] (log_2)	
		NCPPB 3335		Psv-ΔgacA			
		SSM	HIM	SSM	HIM	SSM	HIM
**Upregulated**							
PSA3335_RS19670	Hypothetical protein	28.59	135.22	88.87	827.54	**1.64**	**2.61**
**Downregulated**							
PSA3335_RS01105	Acyl-CoA dehydrogenase	231.85	1556.07	55.31	531.98	**-2.07**	**-1.55**
PSA3335_RS01110	CaiB/BaiF CoA-transferase family protein	145.37	354.13	39.78	167.53	**-1.87**	**-1.08**
PSA3335_RS15805	Transcriptional regulator	166.47	239.36	61.03	91.41	**-1.45**	**-1.39**
PSA3335_RS15815	Excinuclease ABC subunit UvrC	146.90	123.83	4.35	4.42	**-5.07**	**-4.81**
PSA3335_RS20910	1-aminocyclopropane-1-carboxylate deaminase	29.72	42.35	14.14	18.51	**-1.07**	**-1.19**
PSA3335_RS22640	Periplasmic protein	2895.82	2718.44	802.28	932.62	**-1.85**	**-1.54**

[a] Locus tag in the genome of *Pseudomonas savastanoi* pv. savastanoi NCPPB 3335 (accession no. NZ_CP008742.1).

[b] FPKM indicates fragments per kilobase of gene fragments per million of readings, in an RNA-Seq analysis.

[c] Fold change indicates average differential gene expression (log_2 normalized) between the wild-type strain and strain Psv-ΔgacA in SSM and HIM media. Positive and negative fold change reflect an increased or decreased level, respectively, of gene expression in strain Psv-ΔgacA. Cells with grey shading and values in bold indicate genes with a significant differential expression ($q < 0.05$).
