# Supplementary material for: GacA reduces virulence and increases competitiveness in planta in the tumorigenic olive pathogen Pseudomonas savastanoi pv. savastanoi
Source: Front Plant Sci. 2024 Feb 5;15:1347982. doi: 10.3389/fpls.2024.1347982 (PMC10875052; doi:10.3389/fpls.2024.1347982)
Supplement: Supplementary file 11 [file Table_8.pdf]

**Table S8.** Differentially expressed genes (DEGs) related to the redox balance and tolerance to oxidative stress identified by RNA-Seq in *Pseudomonas savastanoi* pv. savastanoi Psv-△gacA.

| Locus tag[a] | Annotation | FPKM[b] | | | | Fold change ($log_2$)[c] | |
|---|---|---|---|---|---|---|---|
| | | NCPPB 3335 | | Psv-△gacA | | | |
| | | SSM | HIM | SSM | HIM | SSM | HIM |
| **NADH-quinone oxidoreductase complex** | | | | | | | |
| PSA3335_RS14105 | Subunit NuoA | 36.05 | 909.26 | 84.51 | 415.95 | **1.23** | **-1.13** |
| PSA3335_RS14100 | Subunit NuoB | 163.19 | 1157.36 | 361.80 | 1415.11 | **1.15** | 0.29 |
| PSA3335_RS14095 | Subunit NuoC/D | 42.25 | 486.88 | 87.41 | 376.01 | **1.05** | -0.37 |
| PSA3335_RS14090 | Subunit NuoE | 63.34 | 569.61 | 121.32 | 329.01 | 0.94 | **-0.79** |
| PSA3335_RS14085 | Subunit NuoF | 73.04 | 494.83 | 120.58 | 406.51 | **0.72** | -0.28 |
| PSA3335_RS14080 | Subunit NuoG | 66.24 | 606.42 | 101.42 | 402.58 | **0.61** | -0.59 |
| PSA3335_RS14075 | Subunit NuoH | 42.57 | 369.26 | 61.70 | 241.23 | 0.54 | -0.61 |
| PSA3335_RS14070 | Subunit NuoI | 52.23 | 467.11 | 77.29 | 324.13 | **0.57** | -0.53 |
| PSA3335_RS14065 | Subunit NuoJ | 41.21 | 452.79 | 57.18 | 234.05 | 0.47 | -0.95 |
| PSA3335_RS14060 | Subunit NuoK | 50.68 | 444.84 | 82.52 | 321.91 | 0.70 | -0.47 |
| PSA3335_RS14055 | Subunit L | 54.79 | 436.16 | 70.91 | 259.70 | 0.37 | **-0.75** |
| PSA3335_RS14050 | Subunit M | 38.03 | 354.55 | 51.57 | 167.58 | 0.44 | **-1.08** |
| PSA3335_RS14045 | Subunit NuoN | 52.98 | 335.16 | 70.67 | 184.54 | 0.42 | **-0.86** |
| **ATP synthase complex** | | | | | | | |
| PSA3335_RS28720 | Epsilon chain | 1222.22 | 1157.67 | 1830.65 | 479.63 | **0.58** | **-1.27** |
| PSA3335_RS28725 | Subunit beta | 2087.02 | 2287.29 | 2943.28 | 841.79 | 0.50 | **-1.44** |
| PSA3335_RS28730 | Subunit gamma | 1454.80 | 1494.66 | 1853.63 | 562.64 | 0.35 | **-1.41** |

| | | | | | | | |
|---|---|---|---|---|---|---|---|
| PSA3335_RS28735 | Subunit alpha | 2084.76 | 1975.35 | 2371.87 | 704.42 | 0.19 | **-1.49** |
| PSA3335_RS28740 | Subunit delta | 1606.10 | 1351.29 | 1780.94 | 517.57 | 0.15 | **-1.38** |
| PSA3335_RS28745 | Subunit B | 3251.80 | 2648.43 | 3459.11 | 1091.62 | 0.09 | **-1.28** |
| PSA3335_RS28750 | Subunit C | 1843.91 | 1964.38 | 1798.16 | 547.48 | -0.04 | **-1.84** |
| PSA3335_RS28755 | Subunit A | 364.11 | 278.21 | 325.07 | 184.93 | -0.16 | **-0.59** |
| **Catalase and Peroxidase** | | | | | | | |
| PSA3335_RS03775 | Catalase | 5.69 | 8.19 | 5.97 | 113.99 | 0.07 | **3.80** |
| PSA3335_RS04865 | Catalase/peroxidase HPI | 97.41 | 249.87 | 110.88 | 608.59 | 0.19 | **1.28** |
| PSA3335_RS13270 | Catalase | 24.50 | 79.50 | 28.19 | 280.96 | 0.20 | **1.82** |
| PSA3335_RS27350 | Catalase HPII | 346.68 | 129.21 | 103.96 | 330.58 | **-1.74** | **1.36** |
| PSA3335_RS07525 | Catalase | 296.55 | 95.66 | 125.03 | 200.28 | **-1.25** | **1.07** |
| PSA3335_RS09710 | Peroxide stress protein YaaA | 170.06 | 78.24 | 157.81 | 246.09 | -0.11 | **1.65** |
| PSA3335_RS09415 | Glutathione peroxidase | 241.94 | 114.96 | 174.64 | 189.55 | -0.47 | **0.72** |
| PSA3335_RS23030 | Glutathione peroxidase | 184.02 | 190.16 | 189.38 | 339.28 | 0.04 | **0.84** |
| **Transcriptional regulators in response to oxidative stress.** | | | | | | | |
| PSA3335_RS17805 | LysR family transcriptional regulator (SoxR) | 65.14 | 324.03 | 118.29 | 104.23 | **0.86** | **-1.64** |
| PSA3335_RS27020 | Hydrogen peroxide-inducible genes activator (OxyR) | 299.05 | 233.94 | 264.91 | 313.98 | -0.17 | 0.42 |
| PSA3335_RS07485 | LysR family transcriptional regulator (FinR) | 27.35 | 47.77 | 29.78 | 34.25 | 0.12 | -0.48 |
| PSA3335_RS00850 | Transcriptional regulator (HexR) | 87.88 | 215.89 | 85.37 | 145.62 | -0.04 | **-0.57** |
| **Other proteins** | | | | | | | |
| PSA3335_RS04980 | Ferric iron uptake transcriptional regulator | 1500.00 | 671.31 | 973.01 | 1016.98 | **-0.62** | **0.60** |
| PSA3335_RS09450 | Thioredoxin-disulfide reductase | 306.82 | 218.39 | 296.68 | 966.82 | -0.05 | **2.15** |
| PSA3335_RS15580 | Alkyl hydroperoxide reductase F | 261.88 | 170.23 | 229.35 | 300.14 | 0.19 | **0.82** |

| | | | | | | | |
|---|---|---|---|---|---|---|---|
| PSA3335_RS00255 | OsmC family peroxiredoxin | 4441.99 | 2275.22 | 1766.50 | 6006.12 | **-1.33** | **1.40** |
| PSA3335_RS06880 | Aquaporin | 32.58 | 83.042 | 25.10 | 336.27 | -0.38 | **2.02** |
| PSA3335_RS12780 | Fumarate/nitrate reduction Fnr | 120.10 | 209.30 | 230.88 | 316.28 | **0.94** | **0.60** |
| PSA3335_RS15575 | Peroxiredoxin | 1624.32 | 722.28 | 1224.03 | 3497.90 | -0.41 | **2.28** |
| PSA3335_RS15150 | Metalloprotease | 192.53 | 173.38 | 173.05 | 291.57 | -0.15 | **0.75** |
| PSA3335_RS15585 | Lactoylglutathion lyase | 1023 | 292.72 | 706.36 | 468.15 | **-0.53** | **0.68** |
| PSA3335_RS00115 | Trk system potassium transporter (CioA) | 35.06 | 74.04 | 42.04 | 44.46 | 0.26 | **-0.74** |
| PSA3335_RS01960 | Peptide-methionine oxide reductase (MsrA) | 320.75 | 436.51 | 342.08 | 413.99 | 0.09 | 0.08 |
| PSA3335_RS15215 | MCE family protein (PqiB) | 176.84 | 130.63 | 118.16 | 77.86 | -0.58 | -0.75 |
| PSA3335_RS01030 | Rubredoxin (RubB) | 65.70 | 140.17 | 61.62 | 156.93 | -0.09 | 0.16 |
| PSA3335_RS05570 | Superoxide dismutase (SodA) | 5195.87 | 8.73 | 4246.77 | 8.54 | -0.29 | -0.03 |
| PSA3335_RS06045 | Superoxide dismutase (SodB) | 165.97 | 827.52 | 132.01 | 1894.34 | -0.33 | **1.19** |
| PSA3335_RS05560 | Class II fumarate hydratase (FumC1) | 2223.79 | 7.81 | 1720.38 | 8.11 | -0.37 | 0.06 |
| PSA3335_RS15510 | Glucose-6-phosphate dehydrogenase (Zwf-1) | 846.27 | 175.39 | 419.88 | 189.85 | **-1.01** | 0.11 |
| PSA3335_RS07490 | Ferredoxin NADP reductase (FpR) | 346.44 | 366.53 | 390.65 | 334.24 | 0.17 | -0.13 |
| PSA3335_RS12900 | Aconitate hydratase (AcnA) | 287.26 | 273.53 | 182.49 | 442.20 | **-0.65** | **0.69** |

[a] Locus tag in the genome of *Pseudomonas savastanoi* pv. savastanoi NCPPB 3335 (accession no. NZ_CP008742.1).

[b] FPKM indicates fragments per kilobase of gene fragments per million of readings, in an RNA-Seq analysis.

[c] Fold change indicates average differential gene expression ($\log_2$ normalized) between the wild-type strain and strain Psv-△gacA in SSM and HIM media. Positive and negative fold change reflect an increased or decreased level, respectively, of gene expression in strain Psv-△gacA. Cells with grey shading and values in bold indicate genes with a significant differential expression ($q < 0.05$).
