# Supplementary material for: GacA reduces virulence and increases competitiveness in planta in the tumorigenic olive pathogen Pseudomonas savastanoi pv. savastanoi
Source: Front Plant Sci. 2024 Feb 5;15:1347982. doi: 10.3389/fpls.2024.1347982 (PMC10875052; doi:10.3389/fpls.2024.1347982)
Supplement: Supplementary file 12 [file Table_9.pdf]

Table S9. Differentially expressed genes (DEGs) encoding T3SS effectors identified by RNA-Seq in *Pseudomonas savastanoi* pv. savastanoi Psv-△gacA.

Locus tag[a]	Annotation	FPKM[b]				Fold change (log_2)[c]	
		NCPPB 3335		Psv-△gacA			
		SSM	HIM	SSM	HIM	SSM	HIM
PSA3335_RS10535	AvrE1	12.76	18.35	11.85	23.91	0.11	0.38
PSA3335_RS01620	AvrPto1	6.55	104.61	8.37	467.04	0.35	**2.16**
PSA3335_RS25235	AvrRpm2	32.66	237.85	34.45	627.77	0.08	**1.40**
PSA3335_RS05460	HopA1	62.23	62.21	66.04	87.49	0.09	0.49
PSA3335_RS10695	HopA2	46.34	75.31	51.95	261.41	0.16	**1.80**
PSA3335_RS10490	HopAA1	11.84	44.14	12.26	245.56	0.05	**2.48**
PSA3335_RS02535	HopAB1	33.25	83.07	32.60	156.93	-0.03	**0.92**
PSA3335_RS24240	HopAE1	12.76	17.61	9.93	57.41	-0.36	**1.71**
PSA3335_A0028	HopAF1-1	94.65	343.50	75.82	392.42	-0.32	0.19
PSA3335_RS14505	HopAH2	61.42	105.23	84.33	97.72	0.46	-0.11
PSA3335_RS24880	HopAJ2	170.19	165.47	163.77	184.23	-0.06	0.15
PSA3335_B0010	HopAO1	375.77	829.56	342.62	1468.50	-0.13	**0.82**
PSA3335_RS12020	HopAO2	66.98	78.12	61.22	142.54	-0.13	**0.87**
PSA3335_RS02305	HopAS1	11.39	17.30	12.92	16.65	0.18	-0.05
PSA3335_RS12045	HopAU1	34.86	51.29	34.92	133.49	0.01	**1.38**
PSA3335_RS20790	HopAZ1	23.14	40.81	24.38	195.03	0.08	**2.26**
PSA3335_RS19200	HopBK1	12.18	8.64	20.45	15.16	0.75	0.81
PSA3335_RS02885	HopBL1	37.69	103.06	212.86	84.63	**2.50**	-0.28
PSA3335_RS03825	HopBL2	161.31	534.39	253.01	647.12	**0.65**	0.28
PSA3335_RS02795	HopBM1	413.13	777.06	555.02	1197.47	0.43	**0.62**
PSA3335_RS28230	HopD1	46.06	83.82	51.82	124.40	0.17	**0.57**
PSA3335_RS21975	HopG1	33.26	52.66	41.59	126.42	0.32	**1.26**
PSA3335_RS24700	HopI1	29.40	64.70	29.74	110.54	0.02	**0.77**
PSA3335_RS10525	HopM1	9.63	20.42	9.68	31.31	0.01	0.62
PSA3335_RS28225	HopQ1	17.47	36.29	18.26	71.26	0.06	**0.97**
PSA3335_RS28250	HopR1	17.99	33.73	19.16	57.93	0.09	**0.78**
PSA3335_RS15915	HopV1	14.42	40.64	13.92	77.73	-0.05	**0.94**

[a] Locus tag in the genome of *Pseudomonas savastanoi* pv. savastanoi NCPPB 3335 (accession no. NZ_CP008742.1).

[b] FPKM indicates fragments per kilobase of gene fragments per million of readings, in an RNA-Seq analysis.

[c] Fold change indicates average differential gene expression (log_2 normalized) between the wild-type strain and strain Psv-△gacA in SSM and HIM media. Positive and negative fold change reflect an increased or decreased level, respectively, of gene expression in strain Psv-△gacA. Cells with grey shading and values in bold indicate genes with a significant differential expression ($q < 0.05$).
