# Supplementary material for: GacA reduces virulence and increases competitiveness in planta in the tumorigenic olive pathogen Pseudomonas savastanoi pv. savastanoi
Source: Front Plant Sci. 2024 Feb 5;15:1347982. doi: 10.3389/fpls.2024.1347982 (PMC10875052; doi:10.3389/fpls.2024.1347982)
Supplement: Supplementary file 13 [file Table_10.pdf]

Table S10. Differentially expressed genes (DEGs) encoding virulence determinants identified by RNA-Seq in *Pseudomonas savastanoi* pv. savastanoi Psv-△gacA.

Category and locus tag[a]	Gene	FPKM[b]				Fold change (log2)[c]	
		NCPPB 3335		Psv-△gacA			
		SSM	HIM	SSM	HIM	SSM	HIM
**WHOP region**							
PSA3335_RS13075	*antA*	2.55	203.63	2.61	447.77	0.03	**1.14**
PSA3335_RS13080	*antB*	1.36	140.94	2.09	241.63	0.62	**0.78**
PSA3335_RS13085	*antC*	2.01	111.78	2.07	202.87	0.04	**0.86**
PSA3335_RS13100	*catA*	3.76	63.14	4.76	113.65	0.34	**0.85**
PSA3335_RS13090	*catB*	5.19	71.02	4.04	129.39	-0.36	**0.87**
PSA3335_RS13095	*catC*	1.09	38.15	0.37	40.55	-1.57	0.09
PSA3335_RS13060	*ipoC*	4.33	4.82	4.14	4.96	-0.06	0.04
PSA3335_RS13055	*ipoB*	5.20	4.94	4.07	5.14	-0.36	0.06
PSA3335_RS13050	*ipoA*	5.54	8.04	5.34	7.68	-0.05	-0.07
PSA3335_RS13045	*dhoB*	4.66	5.28	2.59	5.64	**-0.85**	0.09
PSA3335_RS13040	*dhoA*	5.83	8.54	4.24	10.26	-0.46	0.27
PSA3335_RS13035	*benR*	5.10	26.93	10.12	14.87	**0.99**	**-0.86**
**Indole-3-acetic acid metabolism**							
PSA3335_RS05385	*iaaH1d*	226.58	388.17	334.30	284.61	**0.56**	-0.45
PSA3335_RS05390	*iaaM1d*	410.74	911.16	675.21	790.30	**0.72**	-0.21
PSA3335_RS05400	*matEPsn*	32.90	92.66	45.41	83.46	0.46	-0.15
PSA3335_RS05405	*iaaLPsn*	64.21	120.64	84.47	142.11	0.40	0.24
PSA3335_RS08645	*iaaM2d*	33.98	140.63	47.14	243.14	0.47	**0.79**
PSA3335_RS08650	*iaaH2d*	14.33	23.51	14.86	21.91	0.05	-0.10
PSA3335_RS12170	*aldD*	49.78	28.83	21.06	27.75	**-1.24**	-0.06
PSA3335_RS12925	*aldC*	76.27	46.86	49.59	29.18	**-0.62**	**-0.68**
PSA3335_RS17745	*aldB*	77.40	54.89	52.53	65.60	**-0.56**	0.26
PSA3335_RS26890	*aldA*	83.35	316.02	93.34	265.61	0.16	-0.25
PSA3335_RS28260	*iaaLPsv*	57.63	149.29	74.19	144.11	0.36	-0.05
PSA3335_RS28265	*matEPsv*	19.71	37.63	20.76	32.47	0.07	-0.21
**Cytokinin metabolism**							
PSA3335_A0024	*ptz*	535.67	2728.17	1000.91	2309.27	**0.90**	-0.24
PSPSV_RS00115	*idi*	310.29	206.58	291.16	330.87	-0.09	**0.68**
**Di-GMPc metabolism**							
PSA3335_RS06040	*bifA*	29.48	37.85	32.70	35.67	0.15	-0.09
PSA3335_RS01785	*dgcP*	539.19	424.80	388.37	444.24	-0.47	0.06
**Quorum sensing**							

PSA3335_RS21625	*luxI*	43.75	95.27	54.12	73.35	0.31	-0.38
PSA3335_RS21620	*luxR1*	8.14	25.32	10.85	38.56	0.41	0.61
PSA3335_RS04820	*luxR2*	86.98	128.54	81.78	133.97	-0.09	0.05
PSA3335_RS20605	*luxR3*	155.39	32.28	86.15	71.93	**-0.85**	**1.16**
**Na^+/Ca^{2+} exchange3**							
PSA3335_RS04495	*cneA*	34.67	28.61	22.56	84.88	**-0.62**	**1.57**

[a] Locus tag in the genome of *Pseudomonas savastanoi* pv. savastanoi NCPPB 3335 and its plasmids pPsv48A and pPsv48C (accession no. NZ_CP008742.1, FR820585 and NC_019292, respectively).

[b] FPKM indicates fragments per kilobase of gene fragments per million of readings, in an RNA-Seq analysis.

[c] Fold change indicates average differential gene expression (log_2 normalized) between the wild-type strain and strain Psv-△gacA in SSM and HIM media. Positive and negative fold change reflect an increased or decreased level, respectively, of gene expression in strain Psv-△gacA. Cells with grey shading indicate genes with a significant differential expression ($q < 0.05$).

[d] Alleles *iaaM2* and *iaaH2* do not participate in the biosynthesis of the phytohormone IAA, which depends only on the functionality of alleles *iaaM1* and *iaaH1* (Aragón et al. 2014 FEMS Microbiol Lett 356:184).
