# Supplementary material for: GacA reduces virulence and increases competitiveness in planta in the tumorigenic olive pathogen Pseudomonas savastanoi pv. savastanoi
Source: Front Plant Sci. 2024 Feb 5;15:1347982. doi: 10.3389/fpls.2024.1347982 (PMC10875052; doi:10.3389/fpls.2024.1347982)
Supplement: Supplementary file 14 [file DataSheet_14.docx]

Table S1:
Bullock, W.O., Fernandez, J.M. and Short, J.M. (1987) XLI-Blue a high efficiency plasmid transforming recA Escherichia coli strain with ß-galactosidase selection. BioTechniques, 5, 376-378.

Table S2:
Hoang, T.T., Karkhoff-Schweizer, R.R., Kutchma, A.J. and Schweizer, H.P. (1998). A broad-host-range Flp-FRT recombination system for site-specific excision of chromosomally-located DNA sequences: application for isolation of unmarked Pseudomonas aeruginosa mutants. Gene, 212(1), 77-86. DOI: 10.1016/S0378-1119(98)00130-9
